# Basal Forebrain to Ventral Tegmental Area Glutamatergic Pathway Promotes Emergence from Isoflurane Anesthesia in Mice

**DOI:** 10.1523/JNEUROSCI.0007-25.2025

**Published:** 2025-06-26

**Authors:** Xia-Ting Gong, Zhang-Shu Li, Zhuo-Li Chen, Xin-Cheng Wu, Ling-Yi Shangguan, Zhi-Peng Xu, Li Chen, Changxi Yu, Ping Cai

**Affiliations:** ^1^Department of Pharmacology, School of Pharmacy, Fujian Medical University, Fuzhou, Fujian 350108, China; ^2^Fujian Province Key Laboratory of Environment and Health, School of Public Health, Fujian Medical University, Fuzhou, Fujian 350108, China; ^3^Department of Gastric Surgery, Fujian Medical University Union Hospital, Fuzhou, Fujian 350001, China; ^4^School of Basic Medical Sciences, Fujian Medical University, Fuzhou, Fujian 350108, China; ^5^Fujian Key Laboratory of Drug Target Discovery and Structural and Functional Research, Fujian Medical University, Fuzhou, Fujian 350108, China

**Keywords:** basal forebrain, general anesthesia, isoflurane anesthesia, neural circuit, ventral tegmental area

## Abstract

Recent evidence highlights the importance of glutamatergic neurons in the basal forebrain (BF) in promoting cortical activity; however, whether BF glutamatergic neurons are involved in regulating general anesthesia and the underlying neural circuits remains unclear. Here, we showed that the activity of BF glutamatergic neurons decreased during the induction of isoflurane anesthesia and restored during the emergence in mice. Optogenetic activation of these neurons significantly enhanced cortical activation, accelerated behavioral emergence, and improved physiological indicators in both male and female mice under isoflurane anesthesia. Specifically, activation of BF glutamatergic neurons shortened emergence time from isoflurane anesthesia, decreased isoflurane sensitivity, and increased arousal scores of mice. Moreover, optogenetic activation of BF glutamatergic neurons decreased EEG delta power and burst suppression ratio, while increasing pupil size and respiration rate in mice during isoflurane anesthesia. Similar results were observed during the optogenetic activation of BF glutamatergic terminals in the ventral tegmental area (VTA). Additionally, we found that the activity of BF glutamatergic neurons and VTA glutamatergic neurons synchronously fluctuated during isoflurane anesthesia, and optogenetic activation of BF glutamatergic terminals in the VTA potently increased the calcium signals in VTA glutamatergic neurons during isoflurane anesthesia. Collectively, these findings demonstrate that BF glutamatergic neurons promote emergence from isoflurane anesthesia by activating VTA glutamatergic neurons.

## Significance Statement

General anesthesia is widely used in modern medicine; however, its specific neural mechanisms remain poorly understood. The basal forebrain (BF) is a critical component of the ascending arousal system, and its glutamatergic neurons were implicated in sleep-wake behavior and cortical activity. Here, the study found that optogenetic activation of BF glutamatergic neurons significantly promoted cortical activation, behavioral emergence, and physiological indicators in mice under isoflurane anesthesia. Photostimulation of BF glutamatergic terminals in the ventral tegmental area (VTA) produced similar effects and significantly increased the activity of VTA glutamatergic neurons during isoflurane anesthesia. This study illustrated that BF glutamatergic neurons promote emergence from isoflurane anesthesia through activation of VTA glutamatergic neurons, highlighting a potential target for attenuating anesthesia depth and accelerating anesthesia emergence in clinical settings.

## Introduction

General anesthesia has widespread applications in modern medicine, benefiting millions of patients worldwide every year. Although general anesthesia has been utilized in clinical practice for over a century, the specific neural mechanisms are still poorly understood (Rudolph and Antkowiak, 2004; Brown et al., 2010). The basal forebrain (BF) is a cluster of nerve nuclei located on the ventral side of the ventral ganglion (Peng et al., 2023). Previous studies demonstrated the involvement of the BF in regulating multiple neurobiological behaviors (Ananth et al., 2023), such as movement (Kucinski et al., 2019), learning (Baxter et al., 2013), and reward (Tashakori-Sabzevar and Ward, 2018). Recently, as a key structure of the ascending activating system, the BF is proved to participate in the regulation of general anesthesia (Peng et al., 2023). Optogenetic activation of cholinergic neurons in the BF accelerated emergence from propofol anesthesia (Wang et al., 2021), while inactivation or lesion of these neurons increased the potency of various anesthetics (Luo et al., 2020; Leung and Luo, 2021). Chemogenetic activation of parvalbumin-expressing GABAergic neurons in the BF enhanced the anesthetic effects of propofol and isoflurane, manifesting by shortening anesthetic induction time and prolonging recovery time (Cai et al., 2021).

In addition to cholinergic and GABAergic neurons, the BF also contains a considerable number of glutamatergic neurons, which constitute the largest neuronal population within the BF (Agostinelli et al., 2019). Previous evidence showed that glutamatergic neurons in the BF play a critical role in cortical activation (Anaclet et al., 2015; Xu et al., 2015). Chemogenetic activation of BF glutamatergic neurons induced a significant decrease in electroencephalogram (EEG) delta power during slow wave sleep (Anaclet et al., 2015), while optogenetic activation of these neurons induce a rapid transition from non-rapid eye movement (NREM) sleep to wakefulness in mice (Xu et al., 2015). Besides, the ablation of BF glutamatergic neurons resulted in a significant reduction in the duration of NREM sleep in mice (Peng et al., 2020). Moreover, neuroanatomical results showed that the glutamatergic BF projects to many general anesthesia-related regions, including the ventral tegmental area (VTA; Do et al., 2016). Therefore, we hypothesized that the BF glutamatergic neurons play an important role in regulating general anesthesia.

To test this hypothesis, we first employed fiber photometry and synchronous EEG/electromyogram (EMG) recording to investigate the activity of BF glutamatergic neurons during isoflurane anesthesia. Then we employed an optogenetic approach to explore the role of BF glutamatergic neurons in the induction and emergence from isoflurane anesthesia and determine the downstream target of these neurons in the regulation of isoflurane anesthesia, namely, the VTA. Combining optogenetics and fiber photometry, we further investigated the cell type of VTA neurons mediating the emergence-promoting effect of glutamatergic BF→VTA pathway during isoflurane anesthesia.

## Materials and Methods

### Animals

Vglut2-Cre mice were purchased from the Jackson Laboratory (catalog #016963). Vglut2-Cre mice and negative littermates weighing 22–28 g (8–12 weeks old) were used in this study. Male and female were used in this study. Approximately half of the male and half of the female mice were randomly assigned to groups of a predetermined sample size to minimize the effect of gender difference on experimental results. Animals were housed under a standard environment (at an ambient temperature of 24 ± 0.5°C, relative humidity of 55 ± 5%, and 12 h light/dark cycle (lights on at 7 A.M.)], with *ad libitum* access to water and food. All experimental procedures were approved by the Institutional Animal Care and Use Committee of Fujian Medical University (IACUC FJMU 2024-Y-1792).

### Virus injection, optic fiber implantation, and electrode fixation

Mice were anesthetized with 1.0–2% isoflurane and placed on a stereotaxic apparatus (RWD Life Science). After the disinfection, the skin incision was made along the center line to expose the skull. A small hole was drilled using the skull drill above the target area. Six virus types, namely, AAV-EF1α-DIO-jGCaMP7s (BrainVTA), AAV-hEF1α-DIO-hChR2 (H134R)-mCherry (Taitool), AAV-hSyn-DIO-ChrimsonR-mCherry (BrainVTA), AAV-hSyn-DIO-hM3D(Gq)-mCherry (BrainVTA), hSyn-DIO-hM4D(Gi)-mCherry (Taitool), and AAV-hSyn-DIO-stGtACR2-eGFP (Taitool), were slowly microinjected (30 nl/min) into the BF (AP = 0.00 mm; ML = ±1.40 mm; DV = −5.50 mm) and VTA (AP = −3.40 mm; ML = +0.50 mm; DV = −4.80 mm). The glass pipette was left in injection site for 10 min to ensure that the virus diffused fully and then moved upward by 0.1 mm and withdrawn slowly after 5 min stay. After completion of virus injection, the optical fibers (Inper) for fiber photometry recordings or optogenetic stimulation were implanted 0.2 mm above the virus injection site and secured with dental cement. Subsequently, EEG/EMG recording electrodes were implanted into the skull and trapezius muscle, secured with dental cement. During and after surgery, mice were maintained at 37°C until they recovered.

### Fiber photometry recording

Calcium signals from BF glutamatergic neurons were recorded using a two-color fiber optic recording system (Inper Vista, Inper, China). First, mice were placed in an anesthesia induction chamber (L × W × H = 23 × 10 × 17 cm), which was connected to an anesthesia vaporizer (R580S; RWD Life Science, China). Pure oxygen was delivered at a rate of 1.5 L/min, and the concentration of isoflurane was monitored with an anesthesia monitor (BeneView T5; Mindray, China) with an accuracy of 0.1% throughout the experiment. After the mice were acclimated to the induction chamber, the baseline of calcium signals was recorded for 30 min. Then 1.2% isoflurane was delivered into the chamber, and the calcium signals of mice were recorded for 30 min. After the cessation of isoflurane, the calcium signals were recorded for another 30 min. The average Δ*F*/*F* values were calculated before, during, and after treatments.

We utilized EEG/EMG signals to determine the timepoint when loss of consciousness (LOC) or recovery of consciousness (ROC) occurred and further analyzed the change of calcium signals when LOC or ROC occurred. The timepoint of LOC occurrence is determined by the change of EEG from low amplitude and high frequency to high amplitude and low frequency and the decrease of EMG (Bao et al., 2021). We divided the induction period into three phases, namely, the Pre-anesthesia period (−300 s to 0 s; where 0 s indicates the moment of isoflurane on), before the loss of consciousness period (Pre-LOC, from 0 s to LOC), and after the loss of consciousness period (Post-LOC, from LOC to 600 s). Similarly, the occurrence of ROC is determined by the transformation that EEG signals change from high amplitude and low frequency to low amplitude and high frequency, and EMG signals increase (Bao et al., 2021). We also divided it into three phases, namely, the anesthesia period (−300 s to 0 s; where 0 s indicates the moment of isoflurane off), before recovery of consciousness period (Pre-ROC, from 0 s to ROC), and after recovery of consciousness period (Post-ROC, from ROC to 600 s).

### Estimation of induction and emergence time

The mice were placed in an anesthesia induction chamber filled with 1.4% isoflurane and given 30 Hz photostimulation (3–5 mW, 10 ms; Jiang et al., 2024; Zhang et al., 2025). The chamber was rotated at 15 s intervals until the mice loss of righting reflex (LORR) and then the photostimulation was stopped. The time from isoflurane exposure to LORR was recorded as the time of anesthesia induction (Bao et al., 2021; Li et al., 2022). After isoflurane anesthesia for 30 min, the mice were removed out from the induction chamber gently and placed in supine position. Photostimulation was given until the mice recovery of righting reflex (RORR; Cai et al., 2023b). The time from exposure to air to RORR was recorded as the anesthesia emergence time. In chemogenetic experiments, vehicle or 1.0 mg/kg clozapine-*N*-oxide (CNO) was intraperitoneally injected into the mice 1 h before the experiment (Cai et al., 2023b; Zhang et al., 2025). In all experiments, the body temperature of mice was maintained by placing a 37°C heating pad under the anesthesia induction chamber.

### Sensitivity of isoflurane during photostimulation

The mice were placed in an anesthesia induction chamber filled with 0.5% isoflurane. After 15 min, photostimulation (30 Hz, 10 ms, 60 s) was given and the induction chamber was gently rotated to observe whether the mice showed LORR. If the mice did not show LORR, the isoflurane concentration was raised by 0.1% until the mice showed LORR, and this isoflurane concentration was recorded (Wang et al., 2019; Xia et al., 2024). For RORR experiments, mice were placed in an anesthesia induction chamber filled with 1.4% isoflurane. After 15 min, photostimulation was given and the induction chamber was rotated to observe for RORR. If the mice did not show RORR, the isoflurane concentration was reduced by 0.1% until the mice showed RORR, and this isoflurane concentration was recorded (Xia et al., 2024).

### Arousal scoring during photostimulation

The mice were placed in the anesthesia induction chamber for 5 min and then exposed to 1.4% isoflurane. After 10 min, the concentration of isoflurane was adjusted to 0.6%. This concentration was kept for 30 min and the mice were observed for LORR. If the mice showed behavioral changes, the isoflurane concentration was increased by 0.1% until the mice showed LORR. The mice were then given photostimulation (30 Hz, 10 ms, 60 s; Cai et al., 2023b). The behavioral responses of mice during photostimulation were observed to assess their arousal scores (Li et al., 2022).

### Optogenetic manipulations during 0.8 and 1.2% isoflurane anesthesia

The EEG/EMG electrodes were connected to an amplifier, while the optical fibers were connected to a blue light source. The mice were placed in the anesthesia induction chamber to acclimate for 5 min. Isoflurane was delivered with pure oxygen at a flow rate of 1.5 L/min. The isoflurane concentration in the induction chamber was maintained at 0.8% for EEG spectrum experiments and maintained at 1.2% for burst suppression ratio experiments (Cai et al., 2023b). An acute 30 Hz photostimulation (3–5 mW, 10 ms, 120 s) was given after 30 min isoflurane anesthesia (Li et al., 2024). EEG/EMG signals of mice were recorded throughout the whole experiment, and the temperature of mice was maintained with a heating pad. After the cessation of photostimulation, EEG/EMG signals were recorded for another 10 min.

In chemogenetic experiments, vehicle or 1.0 mg/kg CNO was intraperitoneally injected into the mice 1 h before the experiment. The EEG signals were recorded for 10 min before induction, and 0.8 or 1.2% anesthesia was maintained for 30 min. Then, EEG recordings were stopped 10 min after the cessation of isoflurane anesthesia. For 0.8% isoflurane anesthesia, we analyzed three phases: the period of induction (5 min after the initiation of anesthesia), maintenance (5 min before the cessation of anesthesia), and emergence (5 min after the cessation of anesthesia; Li et al., 2022; Yin et al., 2023). Each band of EEG frequency were defined as follows: delta, 0.5–4.0 Hz; theta, 4.0–7.0 Hz; alpha, 8.0–15.0 Hz; and beta, 16.0–30.0 Hz (Zhang et al., 2025). For 1.2% isoflurane anesthesia, we analyzed the period of anesthesia maintenance (5 min before the cessation of anesthesia).

### Recording and analysis of mice pupil size and respiratory rate

Mice were anesthetized with 1.2% isoflurane and the head was fixed on a stereotaxic apparatus (RWD Life Science, China). After the pupil diameter became stable, the pupil size was recorded for 1 min as the baseline. An acute photostimulation (30 Hz, 10 ms, 60 s) was given for 1 min, and the diameter of pupil was measured for 1 min, respectively, before, during, and after the light stimulation. During the experiment, images of the mice's eyes were captured with a CMOS camera equipped with an infrared LED array. The images were acquired at a rate of 30 Hz and analyzed by an open-source software Bonsai (http://bonsai-rx.org/; Lin et al., 2019). When extracting a black pupil on a gray iris background, the threshold used is adjusted for each session. The pupil diameter was calculated using a binarized pupil image fitted by a least square fit of ellipse (Grujic et al., 2023).

We used a whole-body plethysmograph system (WBP-4M, TOW-INT TECH, China) to monitor the respiratory rate of mice (Hulsmann et al., 2021). The mice were placed in the volume recorder, which was connected to the sensor, an anesthesia vaporizer, and an anesthesia monitor. When the mice breathe, the rise and fall of their thorax cause the inner volume of the volume recorder to change. This volume change is converted into an electrical signal by a pressure transducer and amplifier, and the respiratory curve is displayed on a computer screen after being processed. The graph is processed by software to calculate tidal volume. Then, 1.2% isoflurane was delivered with oxygen at a flow rate of 0.3 L/min. An acute photostimulation (30 Hz, 10 ms, 60 s) was given to the mice after their respiratory waveforms stabilized. The respiratory curve was recorded 1 min before, during, and after photostimulation.

### Statistical analysis

All data are shown as mean ± SEM. The sample size is determined based on the feasibility of the experiment and our previous experience. The mice were randomly grouped, and experimenters were blinded to experimental conditions. Statistical analyses were performed using GraphPad Prism 8.0 (GraphPad Software). Data were tested for normality using Shapiro–Wilk test. Two-tailed unpaired *t* test, paired *t* test, two-tailed Wilcoxon rank-sum test, Mann–Whitney test, one-way or two-way repeated-measures ANOVA followed by Bonferroni’s multiple-comparison tests analysis of variance. Significance was defined as *p* < 0.05.

## Results

### The calcium activity of BF glutamatergic neurons decreases during isoflurane anesthesia

**Figure 1.**
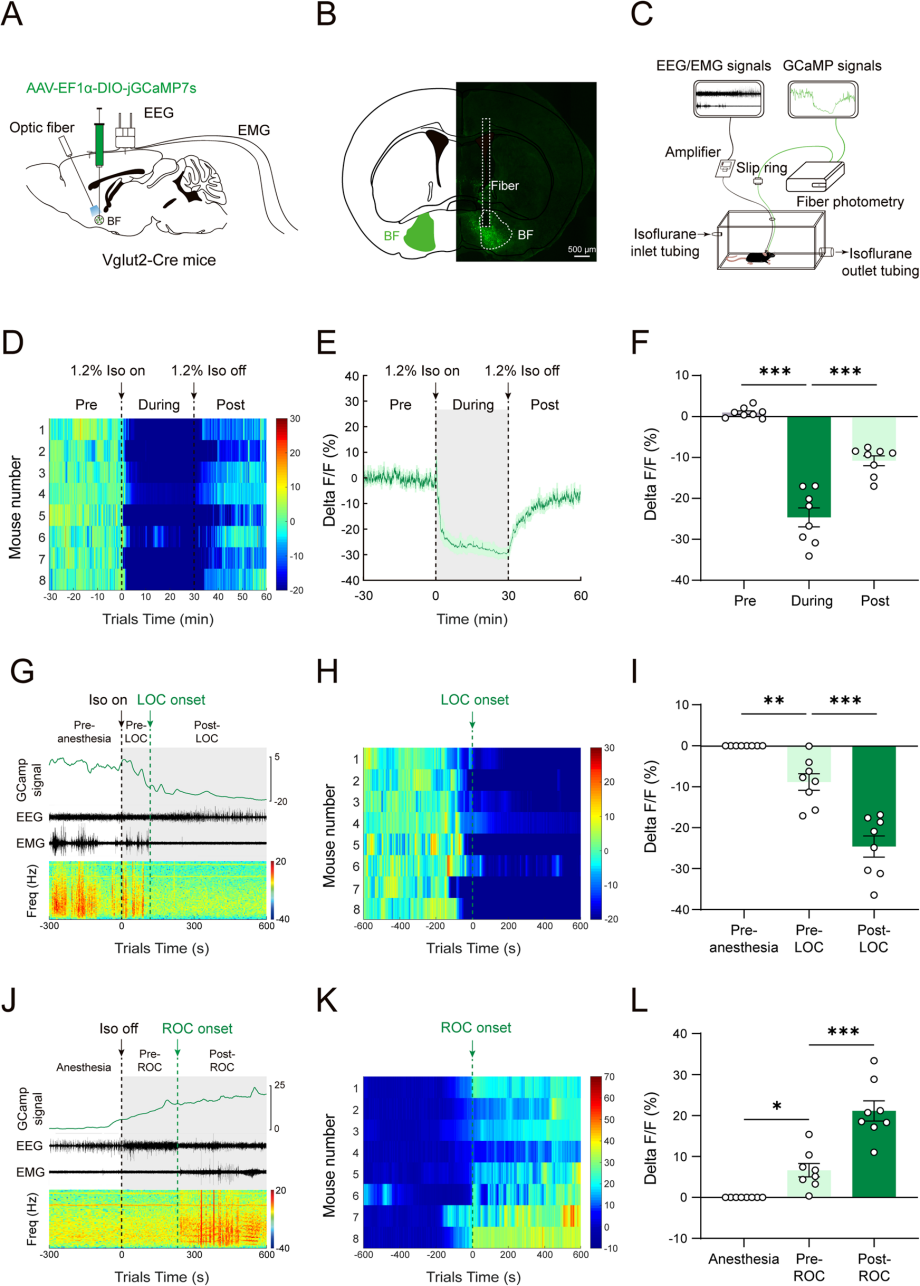
The activity of BF glutamatergic neurons is inhibited during isoflurane anesthesia and restored after isoflurane exposure. ***A***, Schematic of the injection of AAV-EF1α-DIO-jGCaMP7s into the BF of Vglut2-Cre mice, the implantation of an optical fiber over the BF and EEG/EMG implantation. ***B***, Representative image showing the expression of jGCaMP7s in the BF glutamatergic neurons; scale bar, 500 µm. ***C***, Schematic diagram of fiber photometry recording BF glutamatergic neuron activity during isoflurane anesthesia. ***D***, Heatmaps of calcium signals of BF glutamatergic neurons under 1.2% isoflurane. 0 indicates the onset of 1.2% isoflurane. ***E***, Time course of calcium signals before, during, and after isoflurane exposure (thick line indicates the mean and area of shadow indicates SEM, *n* = 8). ***F***, Statistical chart of calcium signal changes before, during, and after inhalation of 1.2% isoflurane (*n* = 8). ***G***, Representative traces of calcium signals aligned with LOC state transitions induced by 1.2% isoflurane. 0 indicates the onset of 1.2% isoflurane. ***H***, Heatmaps of calcium signals of BF glutamatergic neurons during LOC induced by 1.2% isoflurane. 0 indicates the onset of LOC. ***I***, Statistical chart of calcium signal changes in three consecutive time sections during the LOC state transitions (*n* = 8). ***J***, Representative traces of calcium signals aligned with ROC state transitions induced by 1.2% isoflurane. 0 indicates the cessation of 1.2% isoflurane. ***K***, Heatmaps of calcium signals of BF glutamatergic neurons during ROC induced by 1.2% isoflurane. 0 indicates the moment of ROC onset. ***L***, Statistical chart of calcium signal changes in three consecutive time sections during the ROC state transitions (*n* = 8). Asterisks in ***F***, ***I***, and ***L***, indicate significant differences (*p < 0.05, **p < 0.01, ***p < 0.001).

To investigate the correlation of BF glutamatergic neuronal activity with isoflurane anesthesia, we used fiber photometry to record calcium signaling activity in freely moving mice. We injected AAV-EF1α-DIO-jGCaMP7s into the BF of Vglut2-Cre mice, and implanted fiber optics above the BF, and implanted electroencephalography (EEG)/electromyography (EMG) electrodes (Fig. 1*A*). After 4 weeks AAV transfection, jGCaMP7s expression was observed in the BF (Fig. 1*B*). We first examined calcium activity of BF glutamatergic neurons under exposure to 1.2% isoflurane (Fig. 1*C*). The results of fiber photometry recordings showed that calcium signals changed dynamically during isoflurane exposure (Fig. 1*D*,*E*). The calcium signals showed a sharp decline during the initial 100 s after the exposure to 1.2% isoflurane and then showed a slow decrease (Fig. 1*E*). The calcium signals significantly decreased compared with the calcium signals before isoflurane exposure (Pre vs During: 25.58% ± 2.41%, *p* < 0.0001; Fig. 1*F*). After the cessation of isoflurane exposure, the calcium signals gradually restored (During vs Post: −13.85% ± 1.55%, *p* = 0.0001; Fig. 1*F*).

Next, we further analyzed the calcium signals of BF glutamatergic neurons during the induction and emergence periods of 1.2% isoflurane anesthesia according to the previous study (Fig. 1*C*; Bao et al., 2021). We divided the induction phase into three periods: the Pre-anesthesia period (−300 s to 0 s; where 0 s indicates the moment of isoflurane on), Pre-LOC period (the time from 0 s to LOC), and Post-LOC period (the time from LOC to 600 s). Referring to the previous study (Bao et al., 2021), we determined the onset of LOC by the changes in EEG and EMG signals (Fig. 1*G*). The heatmap and statistical results showed that there was a significant decrease in the calcium signals during Pre-LOC period (Pre-anesthesia vs Pre-LOC:8.88% ± 2.03%; *n* = 8, *p* = 0.0097; Fig. 1*H*,*I*) and Post-LOC period (Pre-LOC vs Post-LOC:15.75% ± 1.49%; *n* = 8, *p* < 0.0001; Fig. 1*H*,*I*). For the emergence phase, we also divided it into three periods: the Anesthesia period (−300 to 0 s; where 0 s indicates the moment of isoflurane off), Pre-ROC period (the time from 0 s to ROC), and Post-ROC period (the time from ROC to 600 s). Referring to the previous study (Bao et al., 2021), we determined the onset of ROC by the changes in EEG and EMG signals (Fig. 1*J*). The heatmap and statistical results showed that the calcium signals began to increase in the Pre-ROC period (Anesthesia vs Pre-ROC: −6.63% ± 1.63%; *n* = 8, *p* = 0.0144; Fig. 1*K*,*L*) and continued increasing in Post-ROC period (Pre-ROC vs Post-ROC: −14.51% ± 1.76%; *n* = 8, *p* = 0.0002; Fig. 1*K*,*L*). Taken together, these results indicate that the activity of BF glutamatergic neurons is closely related to isoflurane anesthesia. The significant changes in neuronal activity during LOC and ROC suggest the involvement of BF glutamatergic neurons in isoflurane anesthesia regulation.

### Optogenetic activation of BF glutamatergic neurons promotes behavioral emergence from isoflurane anesthesia

**Figure 2.**
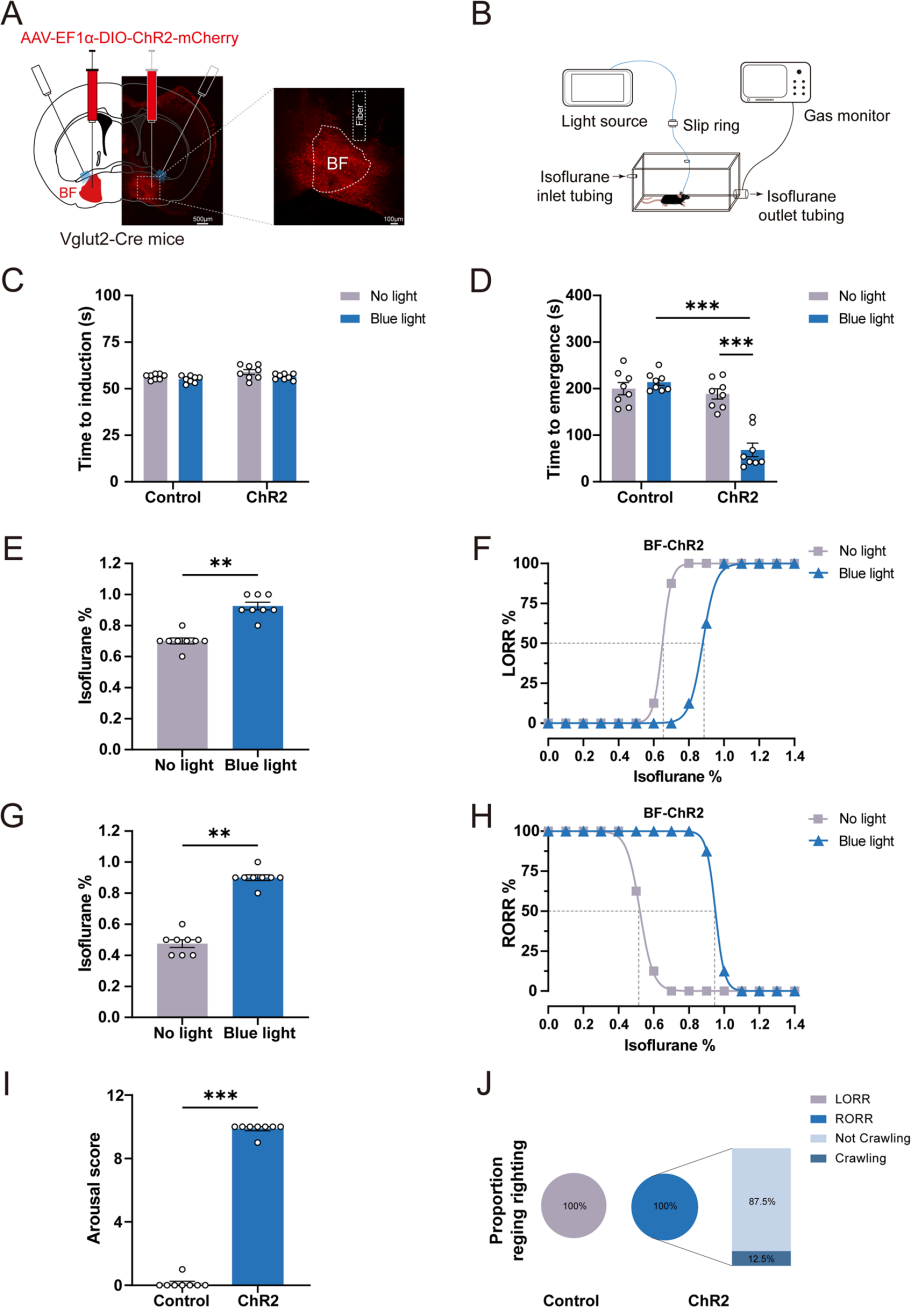
Optogenetic activation of BF glutamatergic neurons facilitates behavioral emergence from isoflurane anesthesia. ***A***, Schematic of the injection of AAV-EF1α-DIO-ChR2-mCherry into the BF of Vglut2-Cre mice and the implantation of an optical fiber over the BF. Representative image of AAV-EF1α-DIO-ChR2-mCherry expression in BF. ***B***, Schematic diagram of optogenetic activation of BF glutamatergic neurons under 1.4% isoflurane anesthesia. ***C***, ***D***, Effect of optogenetic activation of BF glutamatergic neurons on LORR (*n* = 8) and RORR (*n* = 8) time under 1.4% isoflurane anesthesia. ***E***, Isoflurane concentrations inducing LORR with or without optogenetic stimulation of BF glutamatergic neurons in ChR2 mice (*n* = 8). ***F***, Dose–response curves showing the percentage of ChR2 mice experiencing LORR in response to incremental isoflurane concentration with or without optogenetic stimulation of BF glutamatergic neurons (*n* = 8). ***G***, Isoflurane concentrations inducing RORR with or without optogenetic stimulation of BF glutamatergic neurons in ChR2 mice (*n* = 8). ***H***, Dose–response curves showing the percentage of ChR2 mice experiencing RORR in response to decreasing isoflurane concentration with or without optogenetic stimulation of BF glutamatergic neurons (*n* = 8). ***I***, Effect of optogenetic activation of BF glutamatergic neurons on arousal scores in mice under 1.4% isoflurane anesthesia (*n* = 8). ***J***, Pie chart showing the proportion of recovering righting reflex and crawl after optogenetic activation in Control and ChR2 mice (*n* = 8). Additional information to support this figure can be found in Extended Data Figures 2-1–2-3.

To elucidate the regulatory role of BF glutamatergic neurons on isoflurane anesthesia, we used optogenetics to activate BF glutamatergic neurons. The AAV-EF1α-DIO-ChR2-mCherry was injected into the BF of Vglut2-Cre mice, and fiber optics and EEG/EMG electrodes were implanted (Fig. 2*A*). The expression of ChR2-mCherry was observed in the BF after 4 weeks AAV transfection (Fig. 2*A*). Firstly, we investigated the effects of optogenetic activation of BF glutamatergic neurons on anesthesia induction time and emergence time with 1.4% isoflurane (Fig. 2*B*). Our results showed that optogenetic activation of BF glutamatergic neurons did not significantly change induction time but significantly shortened the emergence time in ChR2 mice (No light vs Blue light: from 188.60 ± 10.93 s to 68.38 ± 14.63 s; *n* = 8, *p* < 0.0001; Fig. 2*C*,*D*). In control mice, optogenetic stimulation did not affect the induction time or emergence time (Fig. 2*C*,*D*).

Next, we investigated the effect of optogenetic activation of BF glutamatergic neurons on isoflurane sensitivity. Optogenetic activation of BF glutamatergic neurons required a higher concentration of isoflurane to induce anesthesia in ChR2 mice (No light vs Blue light: from 0.70% ± 0.02% to 0.93% ± 0.03%; *n* = 8, *p* = 0.0078; Fig. 2*E*) and shifted the dose–response curve of LORR to the right and increased the EC_50_ of LORR from 0.65% (95% CI, 0.6496–0.6504%) to 0.88% (95% CI, 0.8745–0.8831%; No light vs Blue light; Fig. 2*F*). Furthermore, optogenetic activation of BF glutamatergic neurons recovered from anesthesia at a higher isoflurane concentration in ChR2 mice (No light vs Blue light: from 0.48% ± 0.03% to 0.90% ± 0.02%; *n* = 8, *p* = 0.0078; Fig. 2*G*), shifted the dose–response curve of RORR to the right, and increased the EC_50_ of RORR from 0.52% (95% CI, 0.5169–0.5255%) to 0.95% (95% CI, 0.9496–0.9504%; No light vs Blue light; Fig. 2*H*). Furthermore, to test the effect of activation of BF glutamatergic neurons on the initiation of anesthesia emergence, we evaluated the arousal scores of mice based on their behavioral responses according to the method described previously (Bao et al., 2021). Our results showed that optogenetic activation of BF glutamatergic neurons potently induced behavioral changes and significantly improved arousal scores in the ChR2 mice, including body (legs, head, and tail) movements (8/8), RORR (8/8), and crawling (7/8; Control vs ChR2: from 0.13 ± 0.13 to 9.88 ± 0.13; *n* = 8, *p* < 0.0001; Fig. 2*I*,*J*; Movie 1; Extended Data Fig. 2-1). There were no obvious behavioral responses during optogenetic stimulation in control mice (Fig. 2*I*,*J*; Movie 2). Taken together, these results indicated that activation of BF glutamatergic neurons accelerated behavioral emergence from isoflurane anesthesia, decreased the sensitivity to isoflurane anesthesia, and facilitated the initiation of anesthesia emergence.

10.1523/JNEUROSCI.0007-25.2025.f2-1 Figure 2-1 The arousal scores after opto-stimulation of BF glutamatergic neurons during isoflurane anesthesia. This table records the details of behavioral response measurements in mice during acute light stimulation (30  Hz, 10  ms, 3-5  mW, 60  s) under isoflurane anesthesia. It records the spontaneous movements of the leg, head, and tail of each mouse, as well as the righting reflex and walking state. The total score for each mouse is obtained by summing up the scores from each category. Download Figure 2-1, DOCX file.

10.1523/JNEUROSCI.0007-25.2025.f2-2 Figure 2-2 **Optogenetic activation of BF glutamatergic neurons facilitates behavioral emergence from isoflurane anesthesia.** (A) -(B) Effect of optogenetic activation (10  Hz, 10  ms) of BF glutamatergic neurons on LORR (n = 7) and RORR (n = 7) time under 1.4% isoflurane anesthesia. (C) Effect of optogenetic activation (10 and 30  Hz, 10  ms) of BF glutamatergic neurons on arousal scores in mice under 1.4% isoflurane anesthesia (n = 7). Download Figure 2-2, TIF file.

**Figure 3.**
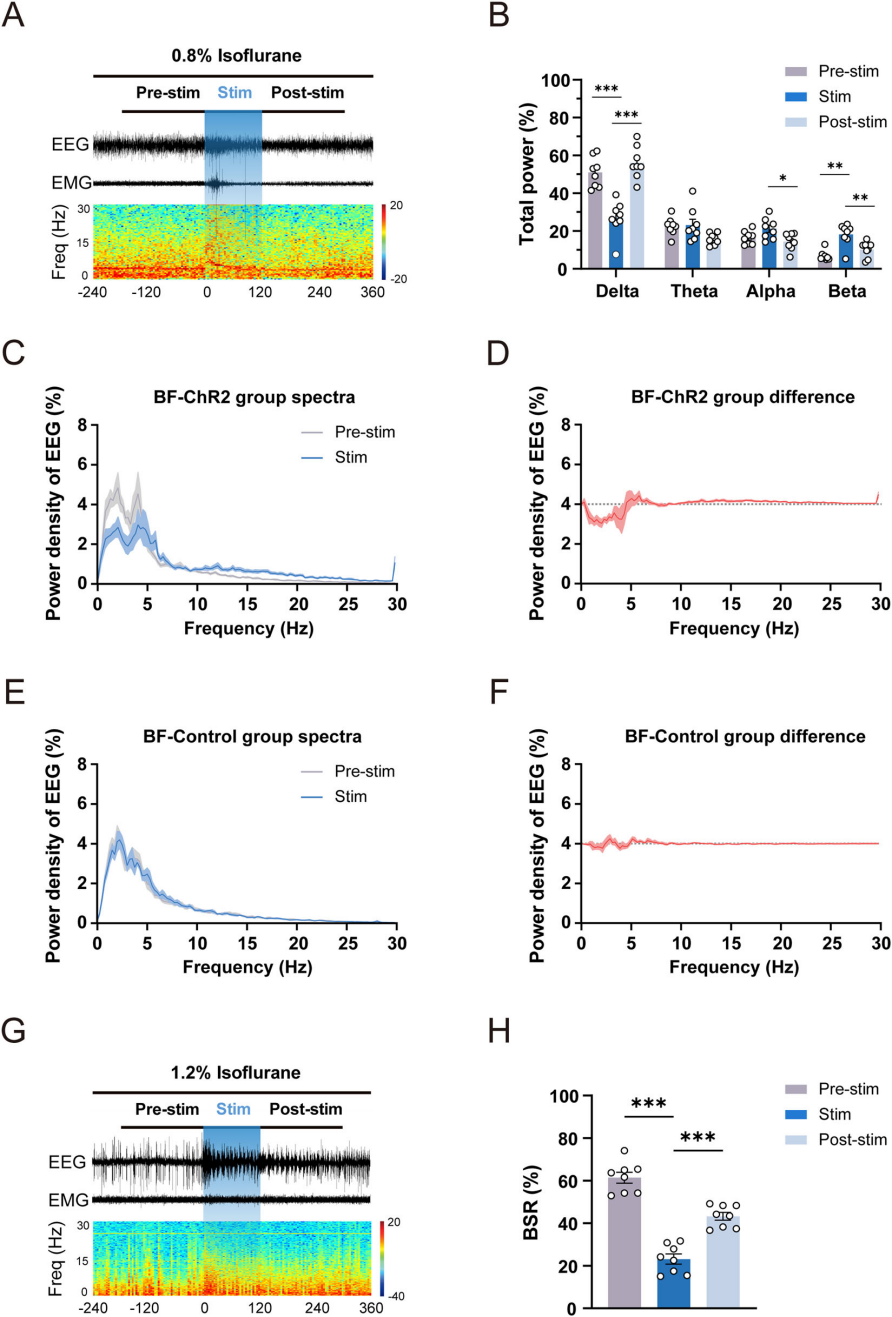
Optogenetic activation of BF glutamatergic neurons promotes cortical activation from isoflurane anesthesia. ***A***, Representative EEG/EMG traces (top) and EEG spectrogram power (bottom) of ChR2 mice before, during, and after optogenetic stimulation (30 Hz, 10 ms, 120 s) under 0.8% isoflurane anesthesia. ***B***, Relative EEG power of ChR2 mice before (gray), during (blue), and after (pale blue) optogenetic stimulation (30 Hz, 10 ms, 120 s) under 0.8% isoflurane anesthesia (*n* = 8). ***C***, Normalized power density of EEG signals of ChR2 mice before and during optogenetic stimulation at 30 Hz (*n* = 8). ***D***, Differences in normalized power density of EEG signals of ChR2 mice before and during optogenetic stimulation at 30 Hz (*n* = 8). ***E***, Normalized power density of EEG signals of control mice before and during optogenetic stimulation at 30 Hz (*n* = 8). ***F***, Differences in normalized power density of EEG signals of control mice before and during optogenetic stimulation at 30 Hz (*n* = 8). ***G***, Representative EEG/EMG traces (top) and EEG spectrogram power (bottom) of ChR2 mice before, during, and after optogenetic stimulation (30 Hz, 10 ms, 120 s) under 1.2% isoflurane anesthesia. ***H***, BSR changes of ChR2 mice before (gray), during (blue), and after (pale blue) optogenetic stimulation (30 Hz, 10 ms, 120 s) under 1.2% isoflurane anesthesia (*n* = 8). Additional information to support this figure can be found in Extended Data Figure 3-1.

10.1523/JNEUROSCI.0007-25.2025.f2-3 Figure 2-3 **The arousal scores after opto-stimulation of BF glutamatergic neurons during isoflurane anesthesia.** This table records the details of behavioral response measurements in mice during yellow or blue light stimulation (10 and 30  Hz, 10  ms, 3-5  mW, 60  s) under isoflurane anesthesia. It records the spontaneous movements of the leg, head, and tail of each mouse, as well as the righting reflex and walking state. The total score for each mouse is obtained by summing up the scores from each category. Download Figure 2-3, DOCX file.

**Movie 1.** Arousal score of BF ChR2 mouse. [View online]

**Movie 2.** Arousal score of BF control mouse. [View online]

### Optogenetic activation of BF glutamatergic neurons enhances cortical activity under isoflurane anesthesia

Cortical EEG could serve as an objective indicator of cortical activity in mice (Cirelli and Tononi, 2015), based on which the depth of anesthesia can be assessed (Cai et al., 2023b). To investigate the role of BF glutamatergic neurons on cortical activity, we first analyzed EEG changes induced by acute optogenetic stimulation (30 Hz, 10 ms, 120 s) with 0.8% isoflurane which make mice at a subnarcotic state. Our results showed that EEG signals exhibited a rapid change from a high-amplitude and low-frequency pattern to a low-amplitude and high-frequency pattern during acute optogenetic stimulation in the ChR2 group (Fig. 3*A*, Movie 3). In the control group, EEG signals did not show obvious changes during acute optogenetic stimulation (Extended Data Fig. 3-1*A,B*; Movie 4). Spectral analysis of EEG signals showed that acute optogenetic stimulation significantly decreased delta power in ChR2 mice (Pre-stim vs stim: 50.98% ± 2.83% to 26.85% ± 3.25%; *n* = 8, *p* = 0.0002), while increased beta power (Pre-stim vs stim: 6.81% ± 0.93% to 18.18% ± 2.07%; *n* = 8, *p* = 0.0016; Fig. 3*B*). To further elucidate the variation of EEG power in different frequency bands, we conducted a detailed analysis of the normalized power density of EEG signals within the 0–30 Hz range. In the ChR2 group, the power density in the low-frequency band (0–4 Hz) was reduced, and the power density in the high-frequency band (10–30 Hz) was increased during photostimulation, comparing with the power density before photostimulation (Fig. 3*C*,*D*). In the control group, photostimulation did not change power density (Fig. 3*E*,*F*).

10.1523/JNEUROSCI.0007-25.2025.f3-1 Figure 3-1 **Optogenetic stimulation of BF glutamatergic neurons does not promotes cortical activation during isoflurane anesthesia in control mice.**
(A) Representative EEG/EMG traces (top) and EEG spectrogram power (bottom) of control mice before, during, and after optogenetic stimulation (30  Hz, 10  ms, 120  s) under 0.8% isoflurane anesthesia.(B) Relative EEG power of control mice before (gray), during (blue), and after (pale blue) optogenetic stimulation (30  Hz, 10  ms, 120  s) under 0.8% isoflurane anesthesia (n = 8).(C) Representative EEG/EMG traces (top) and EEG spectrogram power (bottom) of control mice before, during, and after optogenetic stimulation (30  Hz, 10  ms, 120  s) under 1.2% isoflurane anesthesia.(D) BSR does not changes of control mice before (gray), during (blue), and after (pale blue) optogenetic stimulation (30  Hz, 10  ms, 120  s) under 1.2% isoflurane anesthesia (n = 8). Download Figure 3-1, TIF file.

**Movie 3.** EEG signals of BF ChR2 mouse. [View online]

**Movie 4.** EEG signals of BF control mouse. [View online]

Next, we investigated the effect of acute optogenetic activation (30 Hz, 10 ms, 120 s) of BF glutamatergic neurons at deep anesthesia with 1.2% isoflurane. Burst suppression is a manifestation of severe inhibition of cortical activity (Lewis et al., 2013), and large dosages of anesthetics trigger burst suppression (Kenny et al., 2014). Thus, the burst suppression ratio (BSR) is routinely used as an important index for assessing the depth of anesthesia in animal experiments (Bao et al., 2023a; Cai et al., 2023b). In the ChR2 group, the cortical EEG transited from burst suppression oscillations to a low-voltage fast-activity mode during photostimulation and gradually returned to burst suppression oscillations mode after photostimulation (Fig. 3*G*, Movie 5). In the control group, photostimulation did not change cortical EEG (Extended Data Fig. 3-1*C*,*D*; Movie 6). Statistical results showed that photostimulation significantly reduced BSR in ChR2 group (Pre-stim vs Stim: 61.41% ± 2.58% vs 23.13% ± 2.37%; *n* = 8, *p* < 0.0001; Fig. 3*H*) and the BSR gradually restored after photostimulation (Stim vs Post-stim: 23.13% ± 2.37% vs 43.22% ± 1.84%; *n* = 8, *p* < 0.0001; Fig. 3*H*). These results suggest that optogenetic activation of BF glutamatergic neurons promotes cortical activation and reduces the depth of isoflurane anesthesia. These results suggest that optogenetic activation of BF glutamatergic neurons is sufficient to elicit behavioral arousal during maintenance of shallow anesthesia with isoflurane.

**Movie 5.** BSR of BF ChR2 mouse. [View online]

**Movie 6.** BSR of BF control mouse. [View online]

### Effects of chemogenetic manipulation of BF glutamatergic neurons on behavioral and cortical activity under isoflurane anesthesia

**Figure 4.**
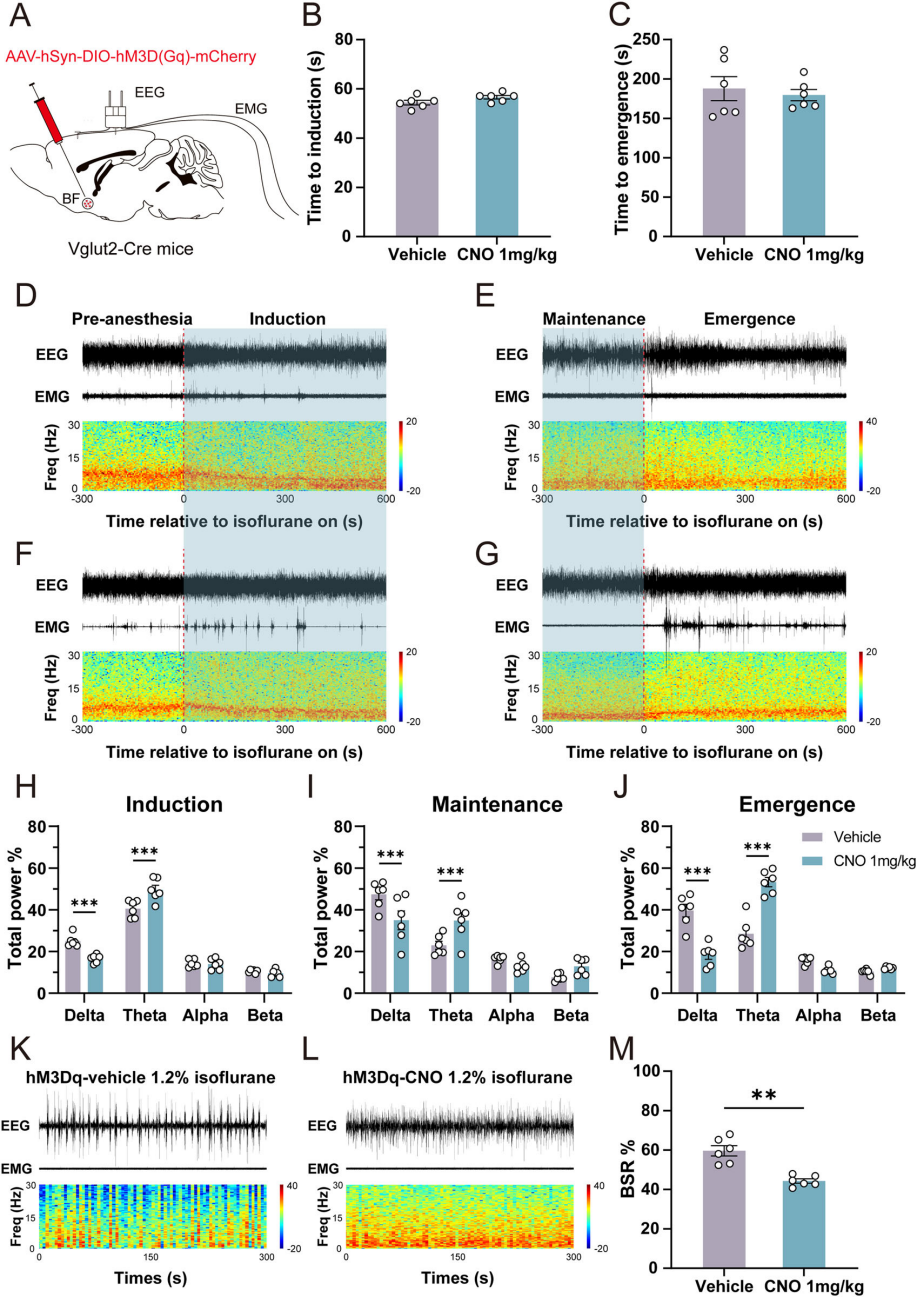
Effects of chemogenetic manipulation of BF glutamatergic neurons on behavioral and cortical activity under isoflurane anesthesia. ***A***, Schematic of the injection of AAV-hSyn-DIO-hM3D(Gq)-mCherry into the BF of Vglut2-Cre mice and the implantation of EEG/EMG electrodes. ***B***, ***C***, Effect of chemogenetic activation of BF glutamatergic neurons on LORR (*n* = 6) and RORR (*n* = 6) time under 1.4% isoflurane anesthesia. ***D***, Representative EEG/EMG traces (top) and EEG spectrogram power (bottom) of hM3D(Gq) mice during isoflurane anesthesia induction period after the administration of vehicle. 0 indicates the onset of 0.8% isoflurane. ***E***, Representative EEG/EMG traces (top) and EEG spectrogram power (bottom) of hM3D(Gq) mice during isoflurane anesthesia emergence period after the administration of vehicle. 0 indicates the cessation of 0.8% isoflurane. ***F***, Representative EEG/EMG traces (top) and EEG spectrogram power (bottom) of hM3D(Gq) mice during isoflurane anesthesia induction period after the administration of 1 mg/kg CNO. ***G***, Representative EEG/EMG traces (top) and EEG spectrogram power (bottom) of hM3D(Gq) mice during isoflurane anesthesia emergence period after the administration of 1 mg/kg CNO. ***H*–*J***, Relative EEG power of hM3D(Gq) mice after the administration of vehicle or 1 mg/kg CNO during 0.8% isoflurane anesthesia induction (***H***), maintenance (***I***), and emergence period (***J***; *n* = 6). ***K***, ***L***, Representative EEG/EMG traces (top) and EEG spectrogram power (bottom) of hM3D(Gq) mice during isoflurane anesthesia after the administration of vehicle (***K***) or 1 mg/kg CNO (***L***). ***M***, BSR changes of hM3D(Gq) mice under 1.2% isoflurane anesthesia after the administration of vehicle or 1 mg/kg CNO (*n* = 6). Additional information to support this figure can be found in Extended Data Figure 4-1.

To further investigate the effects of activating BF glutamatergic neurons on isoflurane anesthesia, we employed chemogenetic techniques to selectively activate these neurons. The AAV-hSyn-DIO-hM3D(Gq)-mCherry was injected into the BF of Vglut2-Cre mice, and EEG/EMG electrodes were implanted (Fig. 4*A*). We first examined the effects of chemogenetic activation of BF glutamatergic neurons on anesthesia induction and emergence times under 1.4% isoflurane. Our results demonstrated that administration of 1 mg/kg CNO did not significantly alter either induction or emergence times (Fig. 4*B*,*C*).

10.1523/JNEUROSCI.0007-25.2025.f4-1 Figure 4-1 **Chemogenetic inhibition of BF glutamatergic neurons did not affect isoflurane anesthesia induction and emergence.**
(A) Schematic of the injection of AAV-hSyn-DIO-hM4D(Gi)-mcherry into the BF of Vglut2-Cre mice and the implantation of EEG/EMG electrodes.(B) Effect of chemogenetic inhibition of BF glutamatergic neurons on LORR time under 1.4% isoflurane anesthesia (n = 6).(C) Effect of chemogenetic inhibition of BF glutamatergic neurons on RORR time under 1.4% isoflurane anesthesia (n = 6).(D) Schematic of the injection of AAV-hSyn-DIO-stGtACR2-eGFP into the BF of Vglut2-Cre mice, the implantation of an optical fiber over the BF and EEG/EMG implantation.(E) Relative EEG power of stGtACR2 mice before (gray), during (blue), and after (pale blue) optogenetic stimulation of BF glutamatergic neurons (20  Hz, 5  ms, 120  s) under 0.8% isoflurane anesthesia (n = 4).(F) BSR does not changes of stGtACR2 mice before (gray), during (blue), and after (pale blue) optogenetic stimulation of BF glutamatergic neurons (20  Hz, 5  ms, 120  s) under 1.2% isoflurane anesthesia (n = 4). Download Figure 4-1, TIF file.

Next, we examined the effect of chemogenetic activation on cortical activity under 0.8% isoflurane anesthesia (Fig. 4*D–G*). Spectral analysis of EEG signals revealed that 1 mg/kg CNO administration significantly decreased delta power (vehicle vs CNO: 25.00% ± 1.18% vs 16.60% ± 0.80%; *n* = 6, *p* = 0.0005; Fig. 4*H*) and increased theta power (vehicle vs CNO: 40.50% ± 1.67% vs 49.56% ± 2.20%; *n* = 6, *p* = 0.0002; Fig. 4*H*) during the anesthesia induction period. During the anesthesia maintenance period, delta power was significantly lower following CNO injection (vehicle vs CNO: 47.39% ± 2.58% vs 34.97% ± 4.51%; *n* = 6, *p* = 0.0001; Fig. 4*I*), accompanied by an increase in theta power (vehicle vs CNO: 22.98% ± 1.92% vs 34.80% ± 3.87%; *n* = 6, *p* = 0.0003; Fig. 4*I*). During the emergence period, CNO administration significantly decreased delta power (vehicle vs CNO: 39.65% ± 3.06% vs 18.40% ± 2.16%; *n* = 6, *p* < 0.0001; Fig. 4*J*) and increased theta power (vehicle vs CNO: 28.47% ± 2.96% vs 53.28% ± 2.23%; *n* = 6, *p* < 0.0001; Fig. 4*J*) compared with vehicle injection. We further investigated the effect of chemogenetic activation of BF glutamatergic neurons on the BSR during 1.2% isoflurane anesthesia. Cortical EEG recordings displayed prominent burst suppression oscillations following vehicle injection (Fig. 4*K*). In contrast, after administration of 1 mg/kg CNO, the cortical EEG shifted to a low-voltage fast-activity mode (Fig. 4*L*). Statistical analysis revealed that CNO injection significantly reduced BSR compared with vehicle injection (vehicle vs CNO: 59.56% ± 2.60% vs 44.25% ± 1.10%; *n* = 6, *p* = 0.0012; Fig. 4*M*).

To investigate whether BF glutamatergic neurons are necessary for emergence from isoflurane anesthesia, we tested the effect of inhibiting these neurons. The inhibitory chemogenetic virus, AAV-hSyn-DIO-hM4D(Gi)-mCherry, was injected into the BF of Vglut2-Cre mice (Extended Data Fig. 4-1*A*). Our results showed that administration of 1 mg/kg CNO did not significantly change either the induction (vehicle vs CNO: 53.50% ± 1.52% vs 55.50% ± 1.65%; *n* = 6, *p* > 0.05; Extended Data Fig. 4-1*B*) or emergence (vehicle vs CNO: 158.20% ± 8.47% vs168.00% ± 6.30%; *n* = 6, *p* > 0.05; Extended Data Fig. 4-1*C*) time under 1.4% isoflurane anesthesia. We also test the effect of optogenetically inhibiting BF glutamatergic neurons. The inhibitory optogenetic virus, AAV-hSyn-DIO-stGtACR2-eGFP, was injected into the BF of Vglut2-Cre mice (Extended Data Fig. 4-1*D*). Firstly, we investigated the effect of optogenetic inhibition of BF glutamatergic neurons on the EEG spectral power during 0.8% isoflurane. Statistical results showed that acute optogenetic stimulation (20 Hz, 5 ms, 120 s) did not significantly change EEG spectral power (Extended Data Fig. 4-1*E*). Next, we investigated the effect of optogenetic inhibition of BF glutamatergic neurons on the BSR under 1.2% isoflurane anesthesia. Statistical results showed that acute optogenetic stimulation (20Hz, 5 ms, 120 s) did not induce obvious BSR change (Pre-stim vs Stim: 64.69% ± 1.43% vs 63.54% ± 6.17%; *n* = 4, *p* > 0.05; Stim vs Post-stim: 63.54% ± 6.17% vs 60.00% ± 3.92%; *n* = 4, *p* > 0.05; Extended Data Fig. 4-1*F*).

### Optogenetic activation of BF glutamatergic neurons elicits pupil dilation and accelerates respiratory rate under isoflurane anesthesia

**Figure 5.**
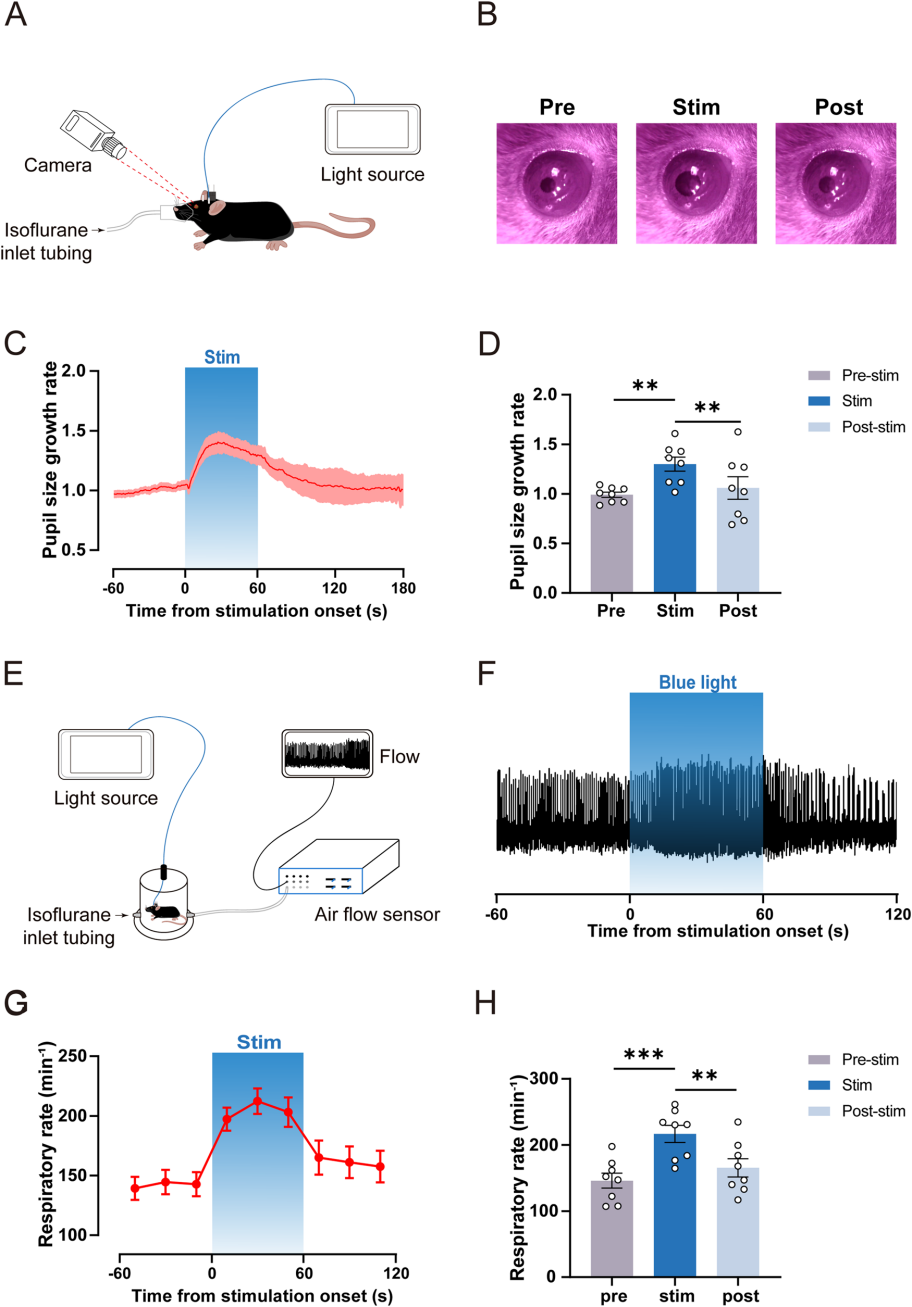
Optogenetic activation of BF glutamatergic neurons elicits pupil dilation and accelerates respiratory rate under isoflurane anesthesia. ***A***, Schematic diagram for measuring pupil size of mice under 1.2% isoflurane anesthesia. ***B***, Representative pupil sizes of ChR2 mice before, during, and after optogenetic activation (30 Hz, 10 ms, 60 s) under isoflurane anesthesia. ***C***, Time course of pupil size of ChR2 mice before, during, and after optogenetic stimulation under 1.2% isoflurane anesthesia (thick line indicates the mean and area of shadow indicates SEM, *n* = 8). ***D***, Statistical chart of pupil changes in ChR2 mice before, during, and after optogenetic stimulation under 1.2% isoflurane anesthesia (*n* = 8). ***E***, Schematic diagram of the measurement of respiration rate in 1.2% isoflurane-anesthetized mice. ***F***, Representative respiratory rates of ChR2 mice before, during, and after optogenetic stimulation (30 Hz, 10 ms, 60 s) under isoflurane anesthesia. ***G***, Time course of respiratory rate of ChR2 mice at 20 s intervals before, during, and after optogenetic stimulation under 1.2% isoflurane anesthesia (*n* = 8). ***H***, Statistical chart of respiratory rate changes in ChR2 mice before, during, and after optogenetic stimulation under 1.2% isoflurane anesthesia (*n* = 8).

The pupil diameter tracks rapid changes in brain state (McGinley et al., 2015) and can be used as an index for characterizing the arousal level in mice (Ren et al., 2024). To further investigate the effect of activating BF glutamatergic neurons on arousal level, we observed the pupil dilation responses elicited by acute optogenetic activation (30 Hz, 10 ms, 60 s) under 1.2% isoflurane anesthesia (Fig. 5*A*). Our results showed photostimulation obviously increased pupil size (Fig. 5*B*, Movie 7). The pupil size rapidly increased within approximately the initial 25 s of photostimulation, maintaining a high level during the later period of photostimulation, and gradually returned to the baseline after the photostimulation (Fig. 5*C*). Statistical results showed acute optogenetic activation of BF glutamatergic neurons caused a significant increase in pupil size (Pre-stim vs Stim: 0.99% ± 0.03% vs 1.30% ± 0.07%; *n* = 8, *p* = 0.0067; Fig. 5*D*), and pupil size recovered after photostimulation (Stim vs Post-stim: 1.30% ± 0.07% vs 1.06% ± 0.11%; *n* = 8, *p* = 0.0064; Fig. 5*D*). In the control group, pupil size did not show obvious changes during acute optogenetic stimulation (Movie 8).

**Movie 7.** Respiratory rate of BF ChR2 mouse. [View online]

**Movie 8.** Respiratory rate of BF control mouse. [View online]

Respiratory rate is also an indication to assess the depth of anesthesia in clinical practice and animal experiments, and it can be utilized as an index for evaluating the depth of anesthesia in mice (Dean et al., 2022). We further investigated whether acute optogenetic activation (30 Hz, 10 ms, 60 s) of BF glutamatergic neurons could affect respiratory rate (Fig. 5*E*). Our results showed that the respiratory movement of mice showed slow wave activity characterized by high tidal volume and low respiratory rate under 1.2% isoflurane anesthesia. The respiratory movement transitioned rapidly from slow wave activity to high respiratory rate during the photostimulation, and the respiratory rate slowly returned to baseline after the cessation of the light stimulation (Fig. 5*F*). Statistical results showed a significant increase in the respiratory rate during photostimulation compared with the rate before photostimulation (Pre-stim vs Stim: 145.80 ± 11.36 vs 216.60 ± 12.89; *n* = 8, *p* < 0.0001; Fig. 5*G*,*H*). After the cessation of the photostimulation, the respiratory rate decreased and slowly recovered (Stim vs Post-stim: 216.60 ± 12.89 vs 165.20 ± 13.84; *n* = 8, *p* = 0.0021; Fig. 5*G*,*H*). These results indicate that optogenetic activation of BF glutamatergic neurons increases pupil size and accelerates respiratory rate of mice under isoflurane anesthesia.

### Optogenetic activation of glutamatergic BF→VTA pathway promotes behavioral emergence from isoflurane anesthesia

**Figure 6.**
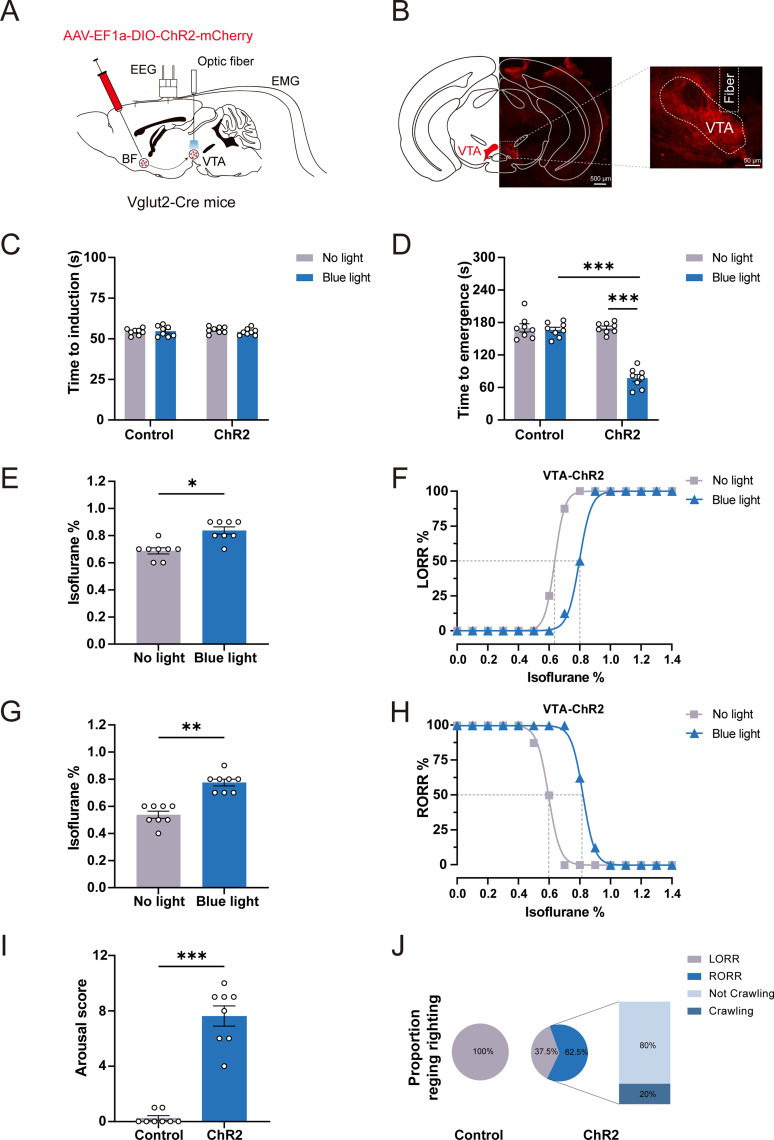
Optogenetic activation of the glutamatergic BF→VTA pathway facilitates behavioral emergence from isoflurane anesthesia. ***A***, Schematic diagram of the optogenetic activation of the glutamatergic BF→VTA pathway. AAV-EF1α-DIO-ChR2-mCherry was injected into the BF of Vglut2-Cre mice, and optic fiber was implanted above the VTA. ***B***, Schematic of a coronal section showing the AAV expression in the VTA and the location of the optical fiber. ***C***, ***D***, Effect of optogenetic activation of the glutamatergic BF→VTA pathway on the LORR (*n* = 8) and RORR (*n* = 8) time of mice under 1.4% isoflurane anesthesia. ***E***, Isoflurane concentrations inducing LORR with or without optogenetic activation of the BF→VTA glutamatergic pathway (*n* = 8) in ChR2 mice. ***F***, Dose–response curves showing the percentage of ChR2 mice experiencing LORR in response to incremental isoflurane concentration with or without optogenetic activation of the BF→VTA glutamatergic pathway (*n* = 8). ***G***, Isoflurane concentrations inducing RORR with or without optogenetic activation of the glutamatergic BF→VTA glutamatergic pathway in ChR2 mice (*n* = 8). ***H***, Dose–response curves showing the percentage of ChR2 mice experiencing RORR in response to decreasing isoflurane concentration with or without optogenetic activation of the BF→VTA glutamatergic pathway (*n* = 8). ***I***, Effect of optogenetic activation of glutamatergic BF→VTA pathway on arousal scores in mice under 1.4% isoflurane anesthesia (*n* = 8). ***J***, Pie chart showing the proportion of recovering righting reflex and crawl after optogenetic activation of control and ChR2 mice (*n* = 8). Additional information to support this figure can be found in Extended Data Figure 6-1.

Previous studies showed that BF glutamatergic neurons innervate multiple brain regions regulating sleep–wakefulness behaviors, such as the medial septum, lateral hypothalamus, lateral habenula, and VTA (Do et al., 2016). Among these structures, the VTA receives abundant projection from the BF. Thus, we speculated that the VTA may be involved in the regulation of isoflurane anesthesia by glutamatergic BF. To test our hypothesis, we injected AAV-EF1α-DIO-ChR2-mCherry into the BF of Vglut2-Cre mice and implanted a fiber optic into the VTA for the manipulation of glutamatergic BF→VTA pathway (Fig. 6*A*). After 4 weeks AAV transfection, obvious expression of ChR2-mCherry and location of fiber optic was observed in VTA (Fig. 6*B*). Similar to the experiments with BF cell soma activation, we firstly investigated the effect of optogenetic activation of glutamatergic BF→VTA pathway on anesthesia induction and emergence time under 1.4% isoflurane. Similar to the effect of BF cell soma activation, optogenetic activation of glutamatergic BF→VTA pathway significantly shortened the emergence time in ChR2 group (No light vs Blue light: from 169.90 ± 3.49 s to 77.25 ± 6.45 s; *n* = 8, *p* < 0.0001; Fig. 6*D*) and did not change isoflurane anesthesia induction time (Fig. 6*C*). These results indicate that optogenetic activation of glutamatergic BF→VTA pathway accelerated behavioral arousal from isoflurane anesthesia.

10.1523/JNEUROSCI.0007-25.2025.f6-1 Figure 6-1 **The arousal scores after opto-stimulation of glutamatergic BF→VTA pathway during isoflurane anesthesia.** This table records the details of behavioral response measurements in mice during acute light stimulation (30  Hz, 10  ms, 3-5  mW, 60  s) under isoflurane anesthesia. It records the spontaneous movements of the leg, head, and tail of each mouse, as well as the righting reflex and walking state. The total score for each mouse is obtained by summing up the scores from each category. Download Figure 6-1, DOCX file.

Similarly, we next investigated the effect of optogenetic activation of glutamatergic BF→VTA pathway on isoflurane sensitivity. The results showed that optogenetic activation of glutamatergic BF→VTA pathway increased the concentration required for LORR (No light vs Blue light: from 0.69% ± 0.02% to 0.84% ± 0.03%; *n* = 8, *p* = 0.0156; Fig. 6*E*) and increased the EC_50_ of LORR from 0.64% (95% CI, 0.6344–0.6377%) to 0.80% (95% CI, 0.7874–0.8036%; No light vs Blue light; Fig. 6*F*). Optogenetic activation of glutamatergic BF→VTA pathway also influenced emergency. It recovered from anesthesia at a higher isoflurane concentration (No light vs Blue light: from 0.54% ± 0.03% to 0.78% ± 0.03%; *n* = 8, *p* = 0.0078; Fig. 6*G*) and increased the EC_50_ of RORR from 0.60% (95% CI, 0.5876–0.6051%) to 0.82% (95% CI, 0.8169–0.8256%; No light vs Blue light; Fig. 6*H*). These results suggest that optogenetic activation of glutamatergic BF→VTA pathway decreases the sensitivity to isoflurane.

Furthermore, we also verified the effect of optogenetic activation of glutamatergic BF→VTA pathway on the initiation of emergence during isoflurane anesthesia. Statistical results showed that optogenetic activation of glutamatergic BF→VTA pathway significantly induced behavioral changes compared with the control group, including body (legs, head, and tail) movements (7/8), RORR (5/8), and crawling (4/8; Control vs ChR2: from 0.25 ± 0.16 to 7.63 ± 0.73; *n* = 8, *p* = 0.0002; Fig. 6*I*,*J*; Extended Data Fig. 6-1). In conclusion, these results suggest that optogenetic activation of glutamatergic BF→VTA pathway is capable of eliciting behavioral arousal during isoflurane anesthesia.

### Optogenetic activation of glutamatergic BF→VTA pathway induced cortical activation in mice during isoflurane anesthesia

**Figure 7.**
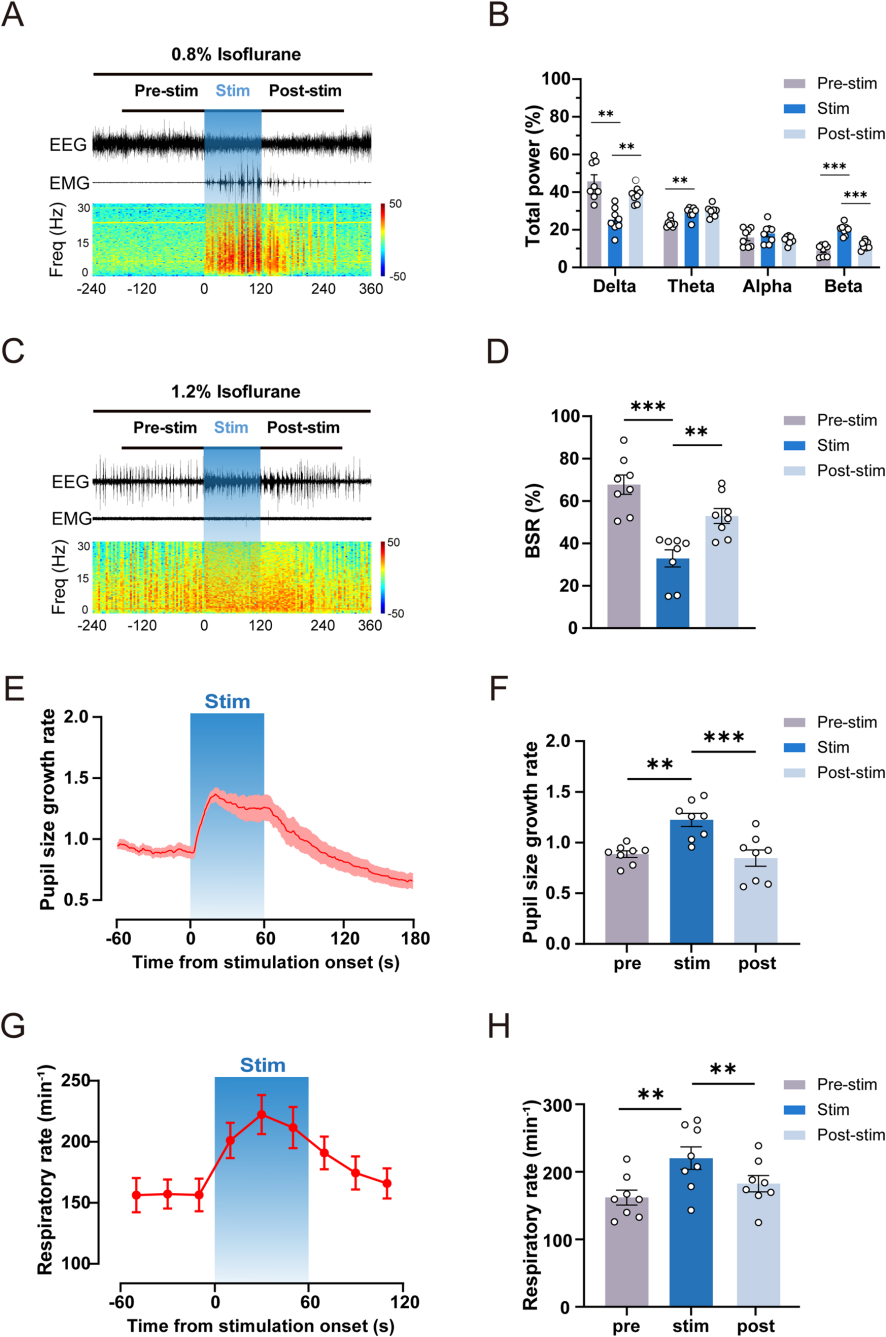
Optogenetic activation of the glutamatergic BF→VTA pathway promotes cortical activation, elicits pupil dilation, and accelerates respiratory rate under isoflurane anesthesia. ***A***, Representative EEG/EMG traces (top) and EEG spectrogram power (bottom) of ChR2 mice before, during, and after optogenetic activation of glutamatergic BF→VTA pathway (30 Hz, 10 ms, 120 s) under 0.8% isoflurane anesthesia. ***B***, Relative EEG power of ChR2 mice before (gray), during (blue), and after (pale blue) optogenetic activation of glutamatergic BF→VTA pathway (30 Hz, 10 ms, 120 s) under 0.8% isoflurane anesthesia (*n* = 8). ***C***, Representative EEG/EMG traces (top) and EEG spectrogram power (bottom) of ChR2 mice before, during, and after optogenetic activation of glutamatergic BF→VTA pathway (30 Hz, 10 ms, 120 s) under 1.2% isoflurane anesthesia. ***D***, BSR changes of ChR2 mice before (gray), during (blue), and after (pale blue) optogenetic activation of glutamatergic BF→VTA pathway (30 Hz, 10 ms, 120 s) under 1.2% isoflurane anesthesia (*n* = 8). ***E***, Time course of pupil size of ChR2 mice before, during, and after optogenetic stimulation under 1.2% isoflurane anesthesia (thick lines indicate the mean and area of shadow indicate SEM, *n* = 8). ***F***, Statistical chart of pupil changes in ChR2 mice before, during, and after optogenetic stimulation under 1.2% isoflurane anesthesia (*n* = 8). ***G***, Time course of respiratory rate of ChR2 mice at 20 s intervals before, during, and after optogenetic stimulation under 1.2% isoflurane anesthesia (*n* = 8). ***H***, Statistical chart of respiratory rate changes in ChR2 mice before, during, and after optogenetic stimulation under 1.2% isoflurane anesthesia (*n* = 8). Additional information to support this figure can be found in Extended Data Figure 7-1.

After verifying the behavioral responses induced by optogenetic activation of glutamatergic BF→VTA pathway, we further investigated its effects on cerebral cortical activity. Similar to the effect of BF cell soma activation, acute optogenetic activation (30 Hz, 10 ms, 120 s) could rapidly induce the brain state of ChR2 mice from slow wave activity or burst suppression mode to low voltage, fast activity (Fig. 7*A*), but not in the control group (Extended Data Fig. 7-1*A,B*). Spectral analysis of EEG data showed that acute optogenetic activation could significantly decrease delta power (Pre-stim vs stim: 45.71% ± 3.51% to 25.30% ± 2.28%; *n* = 8, *p* = 0.0081) while significantly increasing theta power (Pre-stim vs stim: 23.51% ± 0.74% to 29.01 ± 1.05%; *n* = 8, *p* = 0.0040) and beta power (Pre-stim vs stim: 9.06% ± 1.09% to 20.03 ± 1.03%; *n* = 8, *p* < 0.0001; Fig. 7*B*). The corresponding EEG power densities during optogenetic activation for the control and ChR2 groups are shown in Extended Data Figure 7-1*C–F*.

10.1523/JNEUROSCI.0007-25.2025.f7-1 Figure 7-1 **Optogenetic activation of the glutamatergic BF→VTA pathway promotes cortical activation during isoflurane anesthesia.**
(A) Representative EEG/EMG traces (top) and EEG spectrogram power (bottom) of control mice before, during, and after optogenetic stimulation of glutamatergic BF→VTApathway (30  Hz, 10  ms, 120  s) under 0.8% isoflurane anesthesia.(B) Relative EEG power of control mice before (gray), during (blue), and after (pale blue) optogenetic stimulation of glutamatergic BF→VTApathway (30  Hz, 10  ms, 120  s) under 0.8% isoflurane anesthesia (n = 8).(C) Normalized power density of EEG signals of ChR2 mice before and during optogenetic stimulation at 30  Hz (n = 8).(D) Differences in normalized power density of EEG signals of ChR2 mice before and during optogenetic stimulation at 30  Hz (n = 8).(E) Normalized power density of EEG signals of Control mice before and during optogenetic stimulation at 30  Hz (n = 8).(F) Differences in normalized power density of EEG signals of Control mice before and during optogenetic stimulation at 30  Hz (n = 8).(G) Representative EEG/EMG traces (top) and EEG spectrogram power (bottom) of control mice before, during, and after optogenetic stimulation of glutamatergic BF→VTA pathway (30  Hz, 10  ms, 120  s) under 1.2% isoflurane anesthesia.(H) BSR does not changes of control mice before (gray), during (blue), and after (pale blue) optogenetic stimulation of glutamatergic BF→VTA pathway (30  Hz, 10  ms, 120  s) under 1.2% isoflurane anesthesia (n = 8). Download Figure 7-1, TIF file.

Next, we investigated the effect of optogenetic activation of glutamatergic BF→VTA pathway on cortical activity under 1.2% isoflurane anesthesia. The results showed that acute optogenetic activation (30 Hz, 10 ms, 120 s) rapidly interrupted the BSR of EEG and induced low voltage, and BSR gradually recovered after photostimulation (Fig. 7*C*). Statistical results showed that optogenetic activation significantly reduced BSR (Pre-stim vs Stim: 67.71% ± 4.57% vs 32.87% ± 4.03%; *n* = 8, *p* = 0.0007; Fig. 7*D*). After acute optogenetic activation, the BSR gradually recovered (Stim vs Post-stim: 32.87% ± 4.03% vs 52.92% ± 3.57%; *n* = 8, *p* = 0.0017; Fig. 7*D*). Photostimulation did not induce obvious BSR change in the control group (Extended Data Fig. 7-1*G,H*). These results suggest that optogenetic activation of glutamatergic BF→VTA pathway drives cortical activation and reduces the depth of isoflurane anesthesia.

### Optogenetic activation of glutamatergic BF→VTA pathway accelerates respiratory rate and elicits pupil dilation under isoflurane anesthesia

We further investigated the effect of acute optogenetic activation of glutamatergic BF→VTA pathway on pupil dilation responses under 1.2% isoflurane anesthesia. Our results showed that VTA photostimulation (30 Hz, 10 ms, 60 s) increased the pupil diameter (Pre-stim vs Stim: 0.89% ± 0.03% vs 1.22% ± 0.06%; *n* = 8, *p* = 0.0083; Fig. 7*E*,*F*), and pupil diameter returned to the baseline after the photostimulation (Stim vs Post-stim: 1.22% ± 0.06% vs 0.85% ± 0.08%; *n* = 8, *p* < 0.0001; Fig. 7*E*,*F*; Movie 9). In the control group, pupil size did not show obvious changes during acute optogenetic stimulation (Movie 10). Next, we investigated the effect of acute optogenetic activation of glutamatergic BF→VTA pathway on respiratory rate under isoflurane anesthesia. Statistical results showed a significant increase in the respiratory rate during photostimulation of glutamatergic BF→VTA pathway compared with the rate before photostimulation (Pre-stim vs Stim: 161.90 ± 11.03 vs 220.20 ± 16.70; *n* = 8, *p* = 0.0060; Fig. 7*G*,*H*). After photostimulation, the respiratory rate gradually recovered to the baseline (Stim vs Post-stim: 220.20 ± 16.70 vs 182.40 ± 12.06; *n* = 8, *p* = 0.0078; Fig. 7*G*,*H*). These results suggest that optogenetic activation of glutamatergic BF→VTA pathway increased pupil size and respiratory rate under isoflurane anesthesia.

**Movie 9.** Respiratory rate of BF→VTA ChR2 mouse. [View online]

**Movie 10.** Respiratory rate of BF→VTA control mouse. [View online]

### Glutamatergic BF→VTA pathway regulate isoflurane anesthesia through VTA glutamatergic neurons

**Figure 8.**
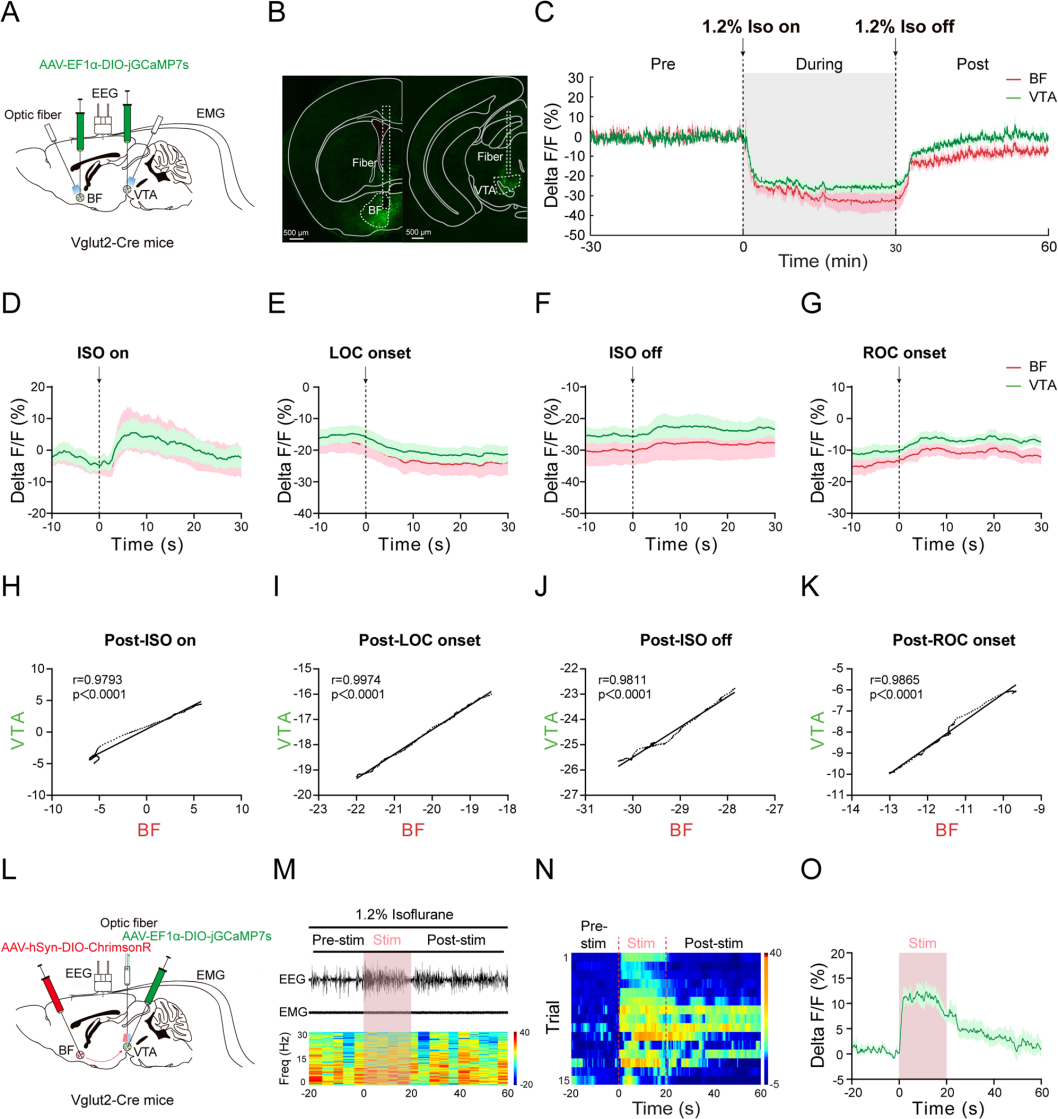
The activity of BF glutamatergic neurons correlates with the activity of VTA glutamatergic neurons and increases calcium signals of VTA glutamatergic neurons during isoflurane anesthesia. ***A***, Schematic of the injection of AAV-EF1α-DIO-jGCaMP7s into the BF and VTA of Vglut2-Cre mice, the implantation of optic fibers over the BF and VTA, and the implantation of EEG/EMG electrodes. ***B***, Representative images showing the expression of jGCaMP7s in the BF and VTA glutamatergic neurons; scale bar, 500 µm. ***C***, Time course of BF (red) and VTA (green) calcium signals before, during, and after isoflurane exposure (thick line indicates the mean and area of shadow indicates SEM, *n* = 8). ***D***–***G***, Time course of BF (red) and VTA (green) calcium signals under different periods, including ISO on (***D***), LOC onset (***E***), ISO off (***F***), and ROC onset (***G***) period (thick line indicates the mean and the area of shadow indicates SEM, *n* = 8). ***H***–***K***, Pearson’s correlation results of 5 s signals in Post-ISO on (***H***), Post-LOC onset (***I***), Post-ISO off (***J***), and Post-ROC onset period (***K***). ***L***, Schematic of the injection of AAV-hSyn-DIO-ChrimsonR-mCherry into the BF, and injection of AAV-EF1α-DIO-jGCaMP7s into the VTA, and implantation of optic fibers over the VTA. ***M***, Representative EEG/EMG traces (top) and EEG spectrogram power (bottom) of mice before, during, and after optogenetic stimulation (30 Hz, 10 ms, 20 s) under 1.2% isoflurane anesthesia. ***N***, Heatmaps of calcium signals of VTA glutamatergic neurons before, during, and after optogenetic stimulation under 1.2% isoflurane anesthesia. 0 indicates the onset of optogenetic stimulation. ***O***, Time course of calcium signals of VTA glutamatergic neurons before, during, and after optogenetic stimulation in 15 trials from 5 mice (thick line indicates the mean and area of shadow indicates SEM, *n* = 15).

A recent study demonstrates that glutamatergic neurons in the VTA play an important role in isoflurane anesthesia (Zhang et al., 2024). We speculated that glutamatergic neurons in the VTA mediate the emergence-promoting effect of the BF→VTA pathway during isoflurane anesthesia. We first investigated the correlation between the activity of BF glutamatergic neurons and VTA glutamatergic neurons during isoflurane anesthesia. AAV-EF1-DIO-jGCaMP7s was injected into the BF and VTA, and fiber optics were implanted above these structures (Fig. 8*A*). After 4 weeks AAV transfection, the expression of jGCaMP7s was observed in the BF and VTA (Fig. 8*B*). Using a multichannel fiber photometry system, we simultaneously examined calcium activity of BF glutamatergic neurons and VTA glutamatergic neurons during 1.2% isoflurane anesthesia. Our results showed that calcium signals of VTA glutamatergic neurons also exhibited an obvious decrease during isoflurane exposure, similar to calcium signals of BF glutamatergic neurons (Fig. 8*C*).

We analyzed the correlation between the activity of BF glutamatergic neurons and VTA glutamatergic neurons during four periods: isoflurane on, LOC, isoflurane off, and ROC periods (Fig. 8*D–G*). The analysis illustrated a strong correlation in calcium signals between BF glutamatergic neurons and VTA glutamatergic neurons during these periods (Fig. 8*H–K*). The statistics show the correlation coefficients of calcium signals between BF glutamatergic neurons and VTA glutamatergic neurons are 0.9793 after turning on isoflurane (*p* < 0.0001; Fig. 8*H*), 0.9974 after LOC onset (*p* < 0.0001; Fig. 8*I*), 0.9811 after turning off isoflurane (*p* < 0.0001; Fig. 8*J*), and 0.9865 after ROC onset (*p* < 0.0001; Fig. 8*K*).

Next, combining optogenetics and fiber photometry, we investigate the effect of activating glutamatergic BF→VTA pathway on the activity of VTA glutamatergic neurons during isoflurane anesthesia. We injected AAV-hSyn-DIO-ChrimsonR-mCherry into the BF and AAV-EF1α-DIO-jGCaMP7s into the VTA. The fiber optic was implanted above the VTA for optogenetic activation and fiber photometry recordings (Fig. 8*L*). Our results showed that an acute red light stimulation of the VTA (30 Hz, 10 ms, 20 s) induced a rapid translation of EEG signals from a high-amplitude and low-frequency pattern to a low-amplitude and high-frequency pattern (Fig. 8*M*). The heatmap showed that the calcium signals of VTA glutamatergic neurons obviously increased during photostimulation period (Fig. 8*N*). Statistics showed that calcium signals of VTA glutamatergic neurons increased by ∼10% after activating BF→VTA pathway (Fig. 8*O*). These results illustrated that VTA glutamatergic neurons mediate the emergence-promoting effect of glutamatergic BF→VTA pathway during isoflurane anesthesia.

## Discussion

The BF is the crucial relay station of the ascending arousal system and has long been implicated in maintaining consciousness and arousal (Weber and Dan, 2016). The BF mainly comprises three neurochemically distinct subtypes: cholinergic, glutamatergic, and GABAergic neurons (Gritti et al., 2006). Previous studies have shown that both cholinergic and GABAergic neurons in the BF facilitate the behavioral and cortical emergence from isoflurane anesthesia in mice (Luo et al., 2018, 2020; Cai et al., 2023b). Similar to the BF cholinergic and GABAergic neurons, our present findings demonstrated that the glutamatergic neurons in the BF also accelerated emergence from isoflurane anesthesia. Notably, different from the activation of GABAergic and cholinergic neurons which significantly prolonged the induction time of isoflurane anesthesia (Gritti et al., 2006; Luo et al., 2018; Cai et al., 2023b), the activation of BF glutamatergic neurons did not significantly change the induction time as shown in current results. Along with the effect of BF glutamatergic neurons on anesthesia, many neural substrates have been shown to mainly influence the emergence from general anesthesia while exerting little effect on the induction period, such as the parabrachial nucleus (Luo et al., 2018) and ventral tegmental area (Zhou et al., 2015). Chemogenetic activation of parabrachial nucleus merely shortened the emergence time but did not prolong the induction time in either propofol or isoﬂurane anesthesia (Luo et al., 2018). Lesions of the ventral tegmental area prolonged the emergence from anesthesia but did little to impact the induction or sensitivity to propofol anesthesia (Zhou et al., 2015). These results, together with our current results, further demonstrated that the emergence and induction period of general anesthesia were not simple inverse neurophysiologic processes, the neural substrates and mechanisms involved in which are quite different. Furthermore, our current results showed that the glutamatergic BF mainly participates in the emergence period from isoflurane anesthesia, rather than the induction process, which represents an ideal target in the regulation of clinical anesthesia. Pharmacological manipulation of BF glutamatergic neurons may provide a potential strategy for accelerating the patients' recovery from general anesthesia without influencing the induction in clinical settings.

Previous results demonstrated that 10 Hz optogenetic stimulation effectively activate BF glutamatergic neurons and produce strong arousal-promoting effects (Xu et al., 2015). In the present study, we employed 30 Hz optogenetic stimulation parameters, considering the differences between general anesthesia and natural sleep (Akeju and Brown, 2017; Yang et al., 2022; Bao et al., 2023b). Sleep is a physiological state in which cortical activity decreases but still maintains a certain level of activity, allowing for a rapid transition from sleep to wakefulness. In contrast, general anesthesia is a pharmacological state characterized by a more pronounced suppression of cortical activity. The EEG patterns under general anesthesia typically exhibit slow waves and burst suppression, which differ markedly from those observed in natural sleep. We also assessed the effects of 10 Hz optogenetic stimulation of BF glutamatergic neurons during isoflurane anesthesia. Similar to 30 Hz stimulation, 10 Hz stimulation significantly increased the arousal score and reduced the time to emergence without affecting the time to induction (Extended Data Fig. 2-2*A–C*). It is worth noting that the effect of 10 Hz optogenetic stimulation on increasing the arousal score is relatively weak. The arousal score induced by 10 Hz stimulation is significantly lower than that induced by 30 Hz stimulation (Blue light 10 Hz vs Blue light 30 Hz: from 1.29 ± 0.42 to 9.00 ± 0.31; *n* = 7, *p* < 0.0001; Extended Data Fig. 2-2*C*, 2-3). Given that 30 Hz optogenetic stimulation is more effective in promoting anesthesia emergence compared with 10 Hz stimulation, we chose to use 30 Hz optogenetic stimulation in the current study.

The VTA contains multiple cell types, including dopaminergic, glutamatergic, and GABAergic neurons (Morales and Margolis, 2017). Previous studies showed that both dopaminergic and glutamatergic neurons in the VTA promoted emergence from general anesthesia (Taylor et al., 2016; Zhang et al., 2024). Optogenetic activation of VTA dopaminergic neurons significantly increased the arousal scores during isoflurane general anesthesia in mice (Taylor et al., 2016). Similarly, optogenetic activation of VTA glutamatergic neurons showed a significant BSR reduction in the EEG of mice after optogenetic activation during isoflurane anesthesia (Zhang et al., 2024). Generally, it is believed that VTA dopaminergic neurons regulate positive emotions, while VTA glutamatergic neurons regulate negative emotions (Morales and Margolis, 2017). Our previous study demonstrated that activation of glutamatergic BF→VTA pathway induced a series of context-dependent defensive behaviors in mice, including escape, fleeing, avoidance, and hiding (Cai et al., 2022). Besides, the latest findings showed that optogenetic activation of the glutamatergic BF→VTA pathway induced anorexia symptoms, including reduced appetite, increased locomotion, and behavioral avoidance in mice (Cai et al., 2023a). Using the transsynaptic tracer wheat germ agglutinin, Cai et al. showed that BF glutamatergic neurons sent direct projections primarily to glutamatergic neurons in the VTA, with a minor portion targeting VTA non-glutamatergic neurons (Cai et al., 2023a). In the current study, we found that the calcium signals of BF glutamatergic neurons synchronized with the calcium signals of VTA glutamatergic neurons during isoflurane anesthesia, and the optogenetic activation of glutamatergic BF→VTA pathway increased the calcium signals of VTA glutamatergic neurons. Based on the results of previous and current studies, we speculated that BF glutamate neurons facilitate emergence from general anesthesia by activating the glutamate neurons, rather than dopamine neurons, in the VTA.

In this study, our results indicate that BF glutamatergic neurons promote anesthesia emergence through their projection to the VTA. Neuroanatomical results indicate that BF glutamatergic neurons project not only to the VTA but also to other downstream targets participating in the regulation of general anesthesia, such as the lateral hypothalamus, lateral habenula (LHb), periaqueductal gray, and dorsal raphe nucleus (Do et al., 2016; Agostinelli et al., 2019). Among these downstream targets, the LHb is recently proved to play a key role in the regulation of general anesthesia. Blocking the output of the LHb greatly reduced the sedative effect of propofol and delayed the induction of propofol administration in mice (Gelegen et al., 2018). Besides, chemogenetic activation of LHb glutamatergic neurons was found to facilitate the induction of isoflurane anesthesia and increase the burst suppression ratio in mice (Liu et al., 2021). The LHb is also regarded as a critical target in regulating general anesthesia and receives a substantial number of fibers involved in general anesthesia. Zhou et al. reported that optogenetic activation of the orexinergic terminals in the LHb facilitated behavioral and cortical emergence and reduced anesthetic depth during sevoflurane anesthesia in mice (Zhou et al., 2023). Zhao et al. showed that optogenetic activation of lateral hypothalamus glutamatergic terminals in the LHb significantly prolonged induction and shorten emergence from isoflurane anesthesia (Zhao et al., 2021). Therefore, we speculated that the glutamatergic BF may also promote anesthesia emergence through its projection to the LHb. Interestingly, previous studies have shown that the glutamatergic BF→LHb pathway integrates sensory cues and modulates emotional responses in mice (Swanson et al., 2022). Swanson et al. demonstrated that the glutamatergic BF→LHb pathway responds to a wide range of sensory cues, including aversive odors, while its optogenetic activation of this pathway drives a strong, reflexive-like aversion in mice (Swanson et al., 2022). Preoperative sensory stimuli—such as intimidating operating room equipment, bright lights, unpleasant odors, or loud noises—can cause tension in patients and decrease patient compliance during anesthetic induction (Kain et al., 2001), thereby increasing anesthetic requirements throughout the perioperative period. Investigating the glutamatergic BF→LHb pathway may provide insights into the neural mechanisms by which sensory stimuli trigger preoperative tension.

Previous clinical research showed that the intraoperative anesthetic requirements were positively correlated with the levels of preoperative anxiety (Yilmaz Inal et al., 2021). Patients with higher baseline anxiety have been shown to consume greater amounts of propofol, sevoflurane, and analgesic consumption to reach appropriate sedation and anesthesia stages, implicating the antianesthetic property of stress and anxiety (Kil et al., 2012; Chen et al., 2022). In the current study, we showed that glutamatergic BF→VTA circuit promotes the emergence from general anesthesia. It is noteworthy that, recent studies have involved this circuit with stress responses, where both glutamatergic neurons in the BF and VTA were proved to respond to stress stimuli and participate in regulating stress-related behaviors (Barbano et al., 2020; Cai et al., 2022, 2023a). Our previous work showed that stressful stimuli robustly increased the calcium signals of BF glutamatergic neurons, such as looming, sound, tail suspension, and predator odors, while photoactivation of these neurons induced avoidance and hiding behavior in mice (Cai et al., 2022). Barbano et al. demonstrated that looming stimulus and predator odors elicited the increased population activity of VTA glutamatergic neurons, while gene ablation and chemogenetic inhibition of VTA glutamatergic neurons abolished the escape behavior induced by stress stimuli (Barbano et al., 2020). The latest study by Cai et al. showed that glutamatergic neurons in the BF and VTA responded to stress stimuli in a correlative manner, and activation of glutamatergic BF→VTA pathway elicited avoidance and anxiety-like behavior in mice (Cai et al., 2023a). These results highlight the importance of glutamatergic BF→VTA circuit in mediating stress-associated responses and anxiety-like behavior. Considering previous results and results in the current study, we speculated that the hyperactivity of the glutamatergic BF→VTA circuit may underlie the higher anesthesia dosage in patients with stress and anxiety. Selective inhibition of glutamatergic neurons in BF and VTA may provide an avenue for reducing anesthesia dosage and advancing anesthetic safety in patients with stress and anxiety.
