# Supplementary material for: Basal Forebrain to Ventral Tegmental Area Glutamatergic Pathway Promotes Emergence from Isoflurane Anesthesia in Mice
Source: J Neurosci. 2025 Jun 26;45(31):e0007252025. doi: 10.1523/JNEUROSCI.0007-25.2025 (PMC12311767; doi:10.1523/JNEUROSCI.0007-25.2025)
Supplement: Figure 2-1 — The arousal scores after opto-stimulation of BF glutamatergic neurons during isoflurane anesthesia. This table records the details of behavioral response measurements in mice during acute light stimulation (30 Hz, 10 ms, 3-5 mW, 60 s) under isoflurane anesthesia. It records the spontaneous movements of the leg, head, and tail of each mouse, as well as the righting reflex and walking state. The total score for each mouse is obtained by summing up the scores from each category. Download Figure 2-1, DOCX file. [file jneuro-45-e0007252025-s001.docx]

| Group | Leg movement | Head movement | Tail movement | Righting | Walking | Total score |
| --- | --- | --- | --- | --- | --- | --- |
| 1-ChR2 | 2 | 2 | 2 | 2 | 2 | 10 |
| 2-ChR2 | 2 | 2 | 2 | 2 | 1 | 9 |
| 3-ChR2 | 2 | 2 | 2 | 2 | 2 | 10 |
| 4-ChR2 | 2 | 2 | 2 | 2 | 2 | 10 |
| 5-ChR2 | 2 | 2 | 2 | 2 | 2 | 10 |
| 6-ChR2 | 2 | 2 | 2 | 2 | 2 | 10 |
| 7-ChR2 | 2 | 2 | 2 | 2 | 2 | 10 |
| 8-ChR2 | 2 | 2 | 2 | 2 | 2 | 10 |
| 1-mCherry | 0 | 0 | 0 | 0 | 0 | 0 |
| 2-mCherry | 0 | 0 | 1 | 0 | 0 | 1 |
| 3-mCherry | 0 | 0 | 0 | 0 | 0 | 0 |
| 4-mCherry | 0 | 0 | 0 | 0 | 0 | 0 |
| 5-mCherry | 0 | 0 | 0 | 0 | 0 | 0 |
| 6-mCherry | 0 | 0 | 0 | 0 | 0 | 0 |
| 7-mCherry | 0 | 0 | 0 | 0 | 0 | 0 |
| 8-mCherry | 0 | 0 | 0 | 0 | 0 | 0 |
