# Supplementary figures and images for: Basal Forebrain to Ventral Tegmental Area Glutamatergic Pathway Promotes Emergence from Isoflurane Anesthesia in Mice
Source: J Neurosci. 2025 Jun 26;45(31):e0007252025. doi: 10.1523/JNEUROSCI.0007-25.2025 (PMC12311767; doi:10.1523/JNEUROSCI.0007-25.2025)

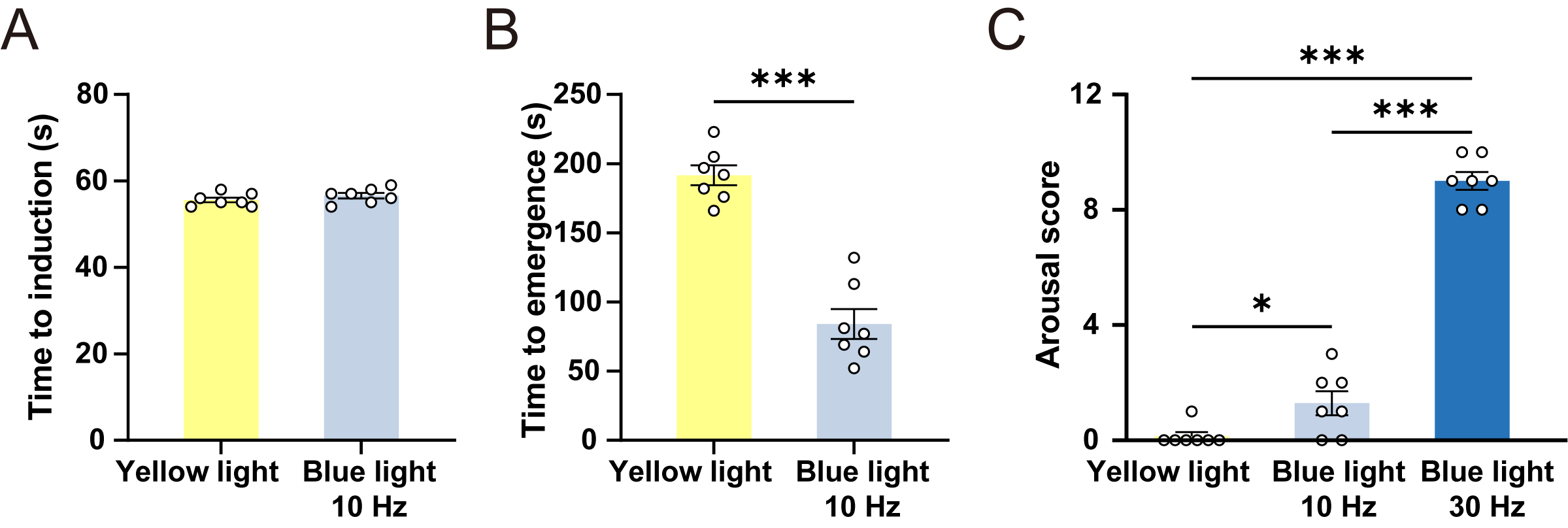

Supplement: Figure 2-2 — Optogenetic activation of BF glutamatergic neurons facilitates behavioral emergence from isoflurane anesthesia. (A) -(B) Effect of optogenetic activation (10 Hz, 10 ms) of BF glutamatergic neurons on LORR (n = 7) and RORR (n = 7) time under 1.4% isoflurane anesthesia. (C) Effect of optogenetic activation (10 and 30 Hz, 10 ms) of BF glutamatergic neurons on arousal scores in mice under 1.4% isoflurane anesthesia (n = 7). Download Figure 2-2, TIF file. [file jneuro-45-e0007252025-s002.tif]

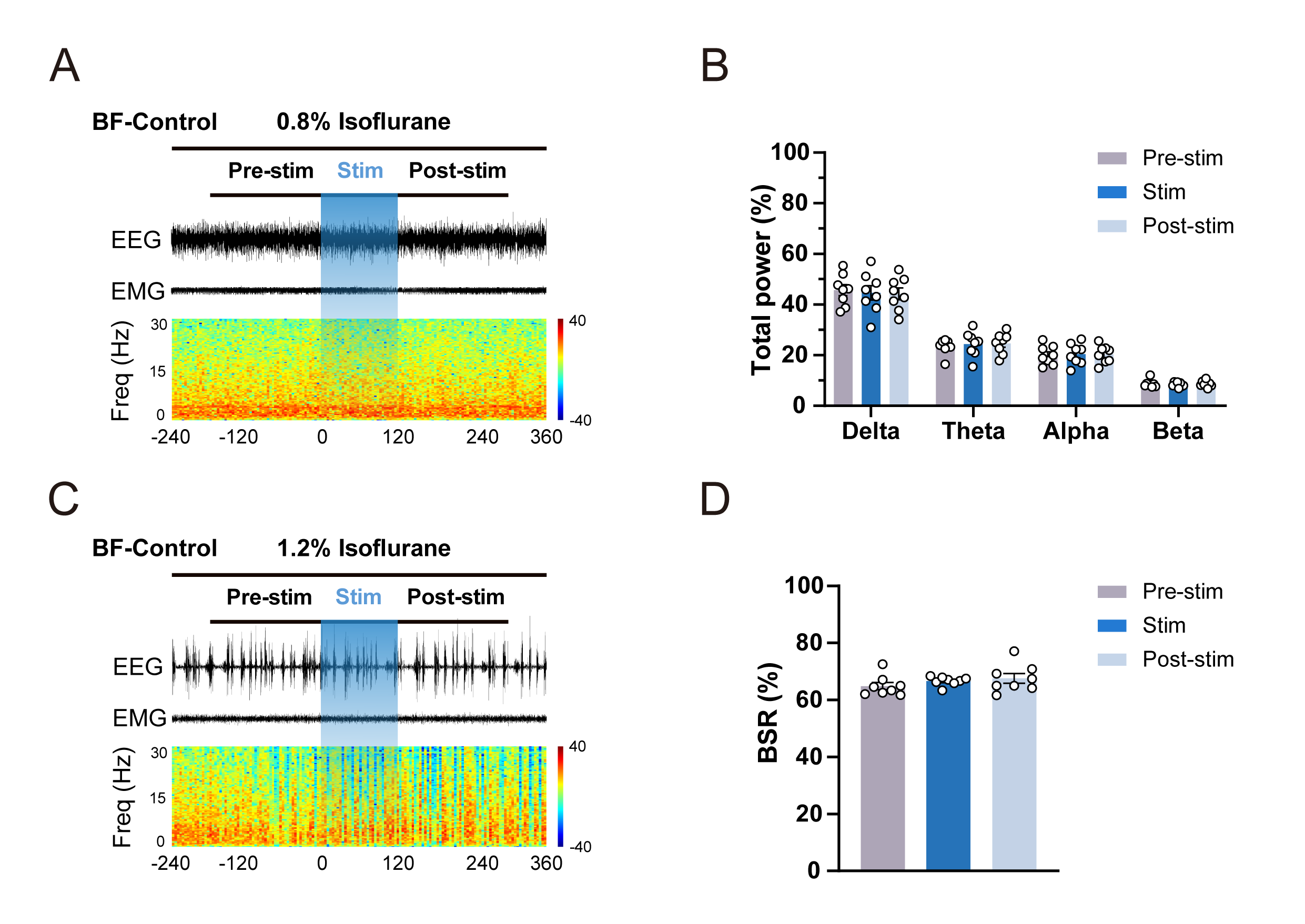

Supplement: Figure 3-1 — Optogenetic stimulation of BF glutamatergic neurons does not promotes cortical activation during isoflurane anesthesia in control mice. (A) Representative EEG/EMG traces (top) and EEG spectrogram power (bottom) of control mice before, during, and after optogenetic stimulation (30 Hz, 10 ms, 120 s) under 0.8% isoflurane anesthesia.(B) Relative EEG power of control mice before (gray), during (blue), and after (pale blue) optogenetic stimulation (30 Hz, 10 ms, 120 s) under 0.8% isoflurane anesthesia (n = 8).(C) Representative EEG/EMG traces (top) and EEG spectrogram power (bottom) of control mice before, during, and after optogenetic stimulation (30 Hz, 10 ms, 120 s) under 1.2% isoflurane anesthesia.(D) BSR does not changes of control mice before (gray), during (blue), and after (pale blue) optogenetic stimulation (30 Hz, 10 ms, 120 s) under 1.2% isoflurane anesthesia (n = 8). Download Figure 3-1, TIF file. [file jneuro-45-e0007252025-s004.tif]

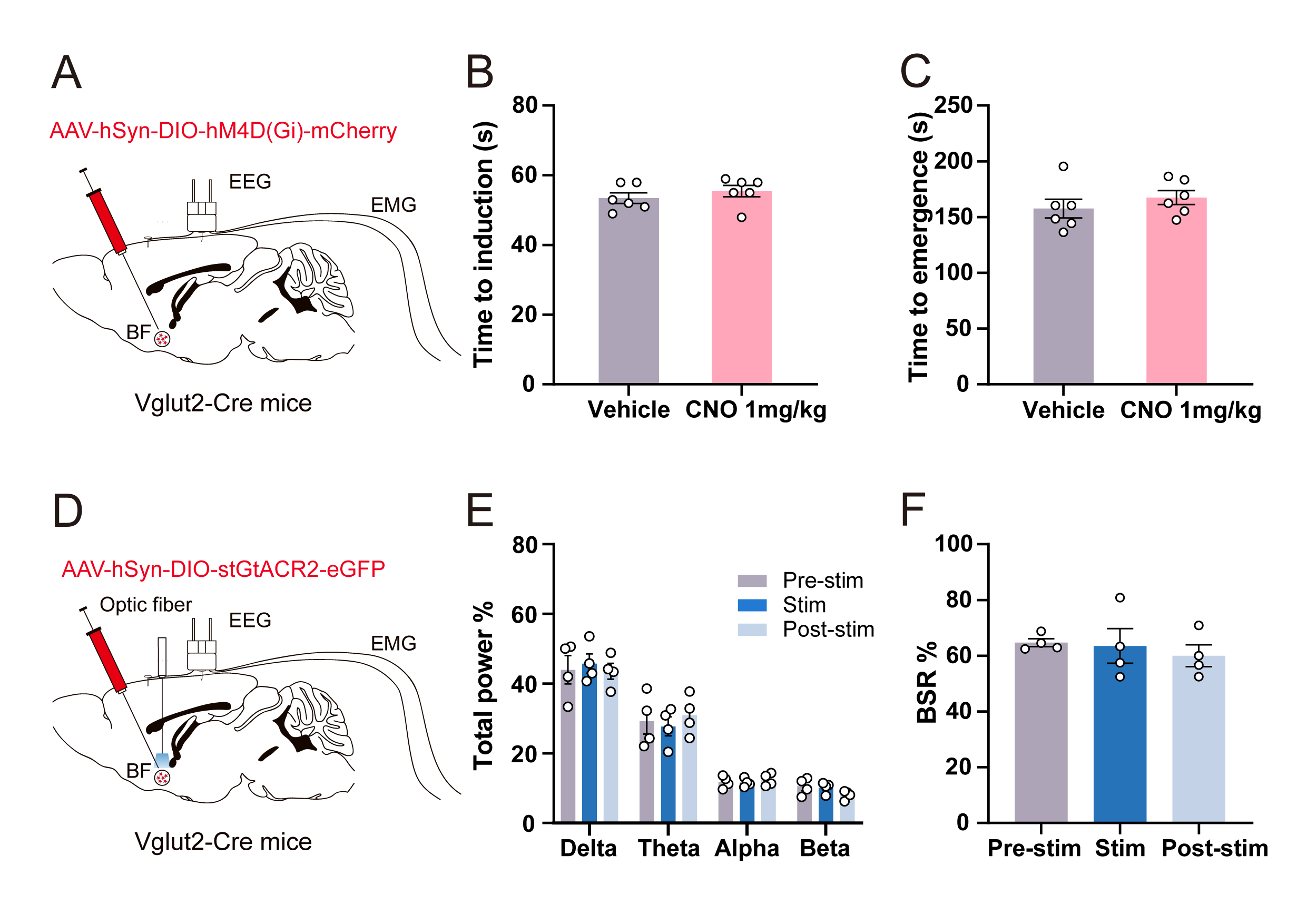

Supplement: Figure 4-1 — Chemogenetic inhibition of BF glutamatergic neurons did not affect isoflurane anesthesia induction and emergence. (A) Schematic of the injection of AAV-hSyn-DIO-hM4D(Gi)-mcherry into the BF of Vglut2-Cre mice and the implantation of EEG/EMG electrodes.(B) Effect of chemogenetic inhibition of BF glutamatergic neurons on LORR time under 1.4% isoflurane anesthesia (n = 6).(C) Effect of chemogenetic inhibition of BF glutamatergic neurons on RORR time under 1.4% isoflurane anesthesia (n = 6).(D) Schematic of the injection of AAV-hSyn-DIO-stGtACR2-eGFP into the BF of Vglut2-Cre mice, the implantation of an optical fiber over the BF and EEG/EMG implantation.(E) Relative EEG power of stGtACR2 mice before (gray), during (blue), and after (pale blue) optogenetic stimulation of BF glutamatergic neurons (20 Hz, 5 ms, 120 s) under 0.8% isoflurane anesthesia (n = 4).(F) BSR does not changes of stGtACR2 mice before (gray), during (blue), and after (pale blue) optogenetic stimulation of BF glutamatergic neurons (20 Hz, 5 ms, 120 s) under 1.2% isoflurane anesthesia (n = 4). Download Figure 4-1, TIF file. [file jneuro-45-e0007252025-s005.tif]

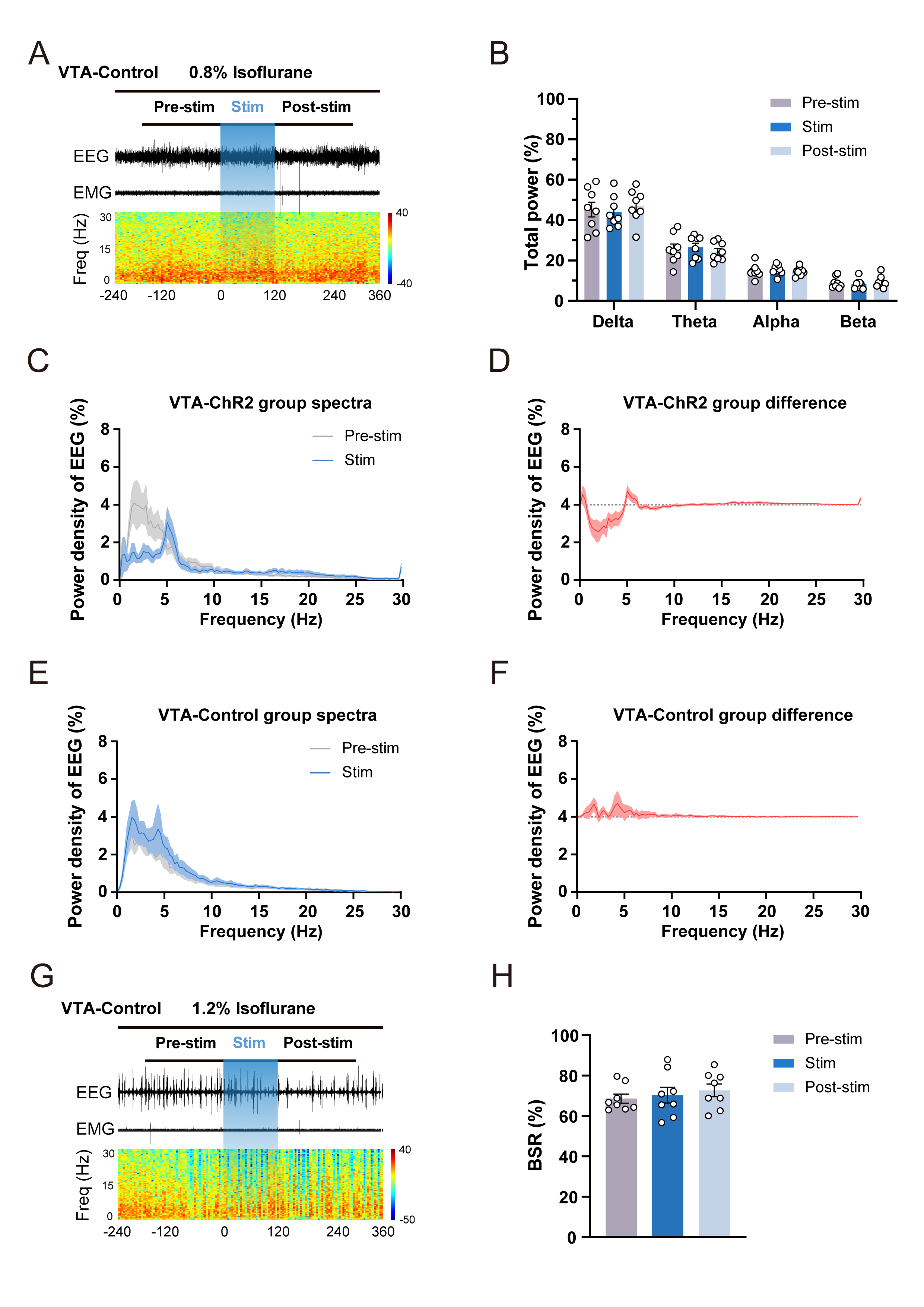

Supplement: Figure 7-1 — Optogenetic activation of the glutamatergic BF→VTA pathway promotes cortical activation during isoflurane anesthesia. (A) Representative EEG/EMG traces (top) and EEG spectrogram power (bottom) of control mice before, during, and after optogenetic stimulation of glutamatergic BF→VTApathway (30 Hz, 10 ms, 120 s) under 0.8% isoflurane anesthesia.(B) Relative EEG power of control mice before (gray), during (blue), and after (pale blue) optogenetic stimulation of glutamatergic BF→VTApathway (30 Hz, 10 ms, 120 s) under 0.8% isoflurane anesthesia (n = 8).(C) Normalized power density of EEG signals of ChR2 mice before and during optogenetic stimulation at 30 Hz (n = 8).(D) Differences in normalized power density of EEG signals of ChR2 mice before and during optogenetic stimulation at 30 Hz (n = 8).(E) Normalized power density of EEG signals of Control mice before and during optogenetic stimulation at 30 Hz (n = 8).(F) Differences in normalized power density of EEG signals of Control mice before and during optogenetic stimulation at 30 Hz (n = 8).(G) Representative EEG/EMG traces (top) and EEG spectrogram power (bottom) of control mice before, during, and after optogenetic stimulation of glutamatergic BF→VTA pathway (30 Hz, 10 ms, 120 s) under 1.2% isoflurane anesthesia.(H) BSR does not changes of control mice before (gray), during (blue), and after (pale blue) optogenetic stimulation of glutamatergic BF→VTA pathway (30 Hz, 10 ms, 120 s) under 1.2% isoflurane anesthesia (n = 8). Download Figure 7-1, TIF file. [file jneuro-45-e0007252025-s007.tif]
